# Cation-induced chirality in a bifunctional metal-organic framework for quantitative enantioselective recognition

**DOI:** 10.1038/s41467-019-13090-9

**Published:** 2019-11-11

**Authors:** Zongsu Han, Kunyu Wang, Yifan Guo, Wenjie Chen, Jiale Zhang, Xinran Zhang, Giuliano Siligardi, Sihai Yang, Zhen Zhou, Pingchuan Sun, Wei Shi, Peng Cheng

**Affiliations:** 1 Key Laboratory of Advanced Energy Materials Chemistry (MOE), College of Chemistry, Nankai University, Tianjin, 300071 China; 2 School of Chemistry, University of Manchester, Manchester, M13 9PL UK; 3 Diamond Light Source, Harwell Science Campus, Didcot, Oxfordshire OX11 0DE UK; 4 School of Materials Science and Engineering, Institute of New Energy Material Chemistry, Nankai University, Tianjin, 300350 China; 5 Key Laboratory of Functional Polymer Materials (MOE), College of Chemistry, Nankai University, Tianjin, 300071 China

**Keywords:** Coordination chemistry, Supramolecular chemistry

## Abstract

The integration of luminescence and chirality in easy-scalable metal-organic frameworks gives rise to the development of advanced luminescent sensors. To date, the synthesis of chiral metal-organic frameworks is poorly predictable and their chirality primarily originates from components that constitute the frameworks. By contrast, the introduction of chirality into the pores of metal-organic frameworks has not been explored to the best of our knowledge. Here, we demonstrate that chirality can be introduced into an anionic Zn-based metal-organic framework via simple cation exchange, yielding dual luminescent centers comprised of the ligand and Tb^3+^ ions, accompanied by a chiral center in the pores. This bifunctional material shows enantioselectivity luminescent sensing for a mixture of stereoisomers, demonstrated for Cinchonine and Cinchonidine epimers and amino alcohol enantiomers, from which the quantitative determination of the stereoisomeric excess has been obtained. This study paves a pathway for the design of multifunctional metal-organic framework systems as a useful method for rapid sensing of chiral molecules.

## Abstract (web summary)

Metal-organic frameworks with luminescence and chirality are promising for luminescent sensors for chiral molecules. Here, the authors use cation exchange to introduce chirality in multifunctional metal-organic framework systems for rapid enantioselective luminescent sensing.

## Introduction

Chirality is crucial to many chemical processes in pharmacy, agriculture, and biology. Enantiomers often display different properties in these processes and, in particular cases, certain isomers can cause fatal effect on living cells^1–3^. Therefore, the recognition of enantiomer is a vital but challenging task. State-of-the-art methods to recognize chiral molecules usually require high-performance liquid chromatography, capillary electrophoresis, gas chromatography, etc. with high running cost and delays in response. By contrast, luminescent sensors have attracted great attention for their easy-operation, low-cost, high efficiency, and ideal portability^4–7^. To construct a chiral luminescent sensor, chiral materials that show selective binding to certain chiral enantiomers are required. Small organic chiral molecules, especially the binaphthyl and its derivatives, and macrocyclic rigid scaffolds have been studied attentively^8–10^. These materials, however, have limited recyclability and are often subject to high cost and synthetic challenges.

The theory and applications of coordination chemistry have been greatly promoted by the research on metal-organic frameworks (MOFs) over the last two decades with their tunable chemical composition and tailored-to-property crystal structures. Due to the designable functionality and porosity of MOFs, a wide range of applications, such as chemical recognition, gas storage and separation, and catalysis, have been studied^11–15^. Small molecules such as volatile organic compounds (VOCs), persistent organic pollutants (POPs) and large molecules such as biomarkers and, in exceptional cases, chiral molecules can be recognized by designed MOFs via luminescence sensing which is facile for operation. Compared with other luminescence materials, porous materials have the ability of adsorbing molecules into the pores achieving local enrichment^16–19^. However, the recognition of chiral molecules remains a highly challenging task due to the similar interactions of the enantiomers with the MOF hosts.

To exhibit the function of chiral recognition and discrimination, MOFs have to be chiral. Currently, there are three main approaches to construct chiral MOFs: (i) direct synthesis using chiral ligands, (ii) chiral-template synthesis, and (iii) post-synthetic chiralization^20–23^. All these methods introduce the chirality to the frameworks of MOFs and usually rely on the use of complex chiral ligands that require multi-step synthesis and purification. As a promising alternative, the introduction of chirality to the pores of MOFs could be easily conducted via exchanging the guest molecules or counter ions with chiral molecules or ions^24–26^, which to our knowledge has remained unexplored to date. On the other hand, although numerous chiral MOFs containing luminescent centers have been reported, very few of them can be used as sensors for enantioselective recognition because of the very small difference of host-guest interactions between the enantiomer and the MOF host^27–31^. The introduction of additional chiral binding sites could effectively overcome this problem^32^.

**Fig. 1:**
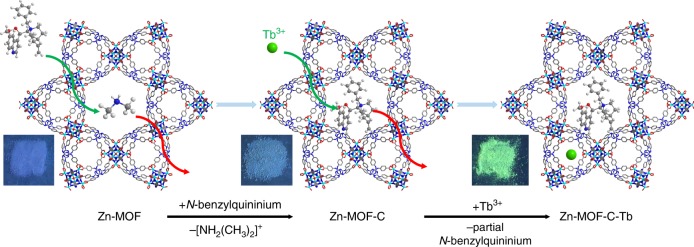
Ion exchange of Zn-MOF. First step: the replacement of (CH_3_)_2_NH_2_^+^ with *N*-benzylquininium cations. Second step: the lead-in of minute quantities of Tb^3+^. The inserted photos were taken under 254 nm irradiation with Xe ultraviolet lamp. Green ball represents Tb, blue for N, gray for C, white for H and turquoise for Zn

Here, we report the introduction of a commercial optically pure compound, *N*-benzylquininium chloride, with five chiral sites into a luminescent Zn-MOF ([(CH_3_)_2_NH_2_]_1/2_[Zn_2_(adenine)(TATAB)O_1/4_]·6DMF·4H_2_O, H_3_TATAB = 4,4′,4″-*s*-triazine-1,3,5-triyltri-*p*-aminobenzoic acid) that contains dimethylamine cations in the one-dimensional hexagonal mesoporous channels^33^, to generate Zn-MOF-C, which exhibits targeted chirality. We then introduced Tb^3+^ as the second luminescent center into the channels of Zn-MOF-C to produce Zn-MOF-C-Tb (Fig. 1 and Supplementary Fig. 1). Importantly, this chiral and luminescent bifunctional MOF with dual luminescent centers has enabled the quantitative enantioselective recognition of chiral molecules for the first time, demonstrated by the epimers of Cinchonine and Cinchonidine, which are potent antimalarial drugs with different half lethal dose and also used as asymmetric catalytic agents^34,35^. Thanks to the dual luminescence from both the ligand and Tb^3+^, the enantiomeric excess (*ee*) value of the epimers can be determined based upon the ratio of luminescence from two centers. Zn-MOF-C-Tb has shown general applicability towards a range of epimers and enantiomers (e.g., amino alcohol) with excellent stability and reusability.

## Results

### Structure and basic characterization

*N*-benzylquininium chloride is widely used as a chiral phase transfer catalyst with five different chiral centers. The framework of Zn-MOF in the solutions of *N*-benzylquininium chloride and TbCl_3_ was stable as confirmed by the powder X-ray diffraction (PXRD) data (Supplementary Fig. 2). Solid-state circular dichroism (CD) spectra of Zn-MOF and Zn-MOF-C showed neutral and negative Cotton effects, respectively, confirming the incorporation of *N*-benzylquininium cations in Zn-MOF-C (Supplementary Fig. 3). The exchange of dimethylamine by *N*-benzylquininium was also confirmed by ^1^H nuclear magnetic resonance (^1^H NMR) spectra as the peaks of the dimethylamine disappeared post the exchange process (Supplementary Fig. 4a and b). Multiple Zn-MOF-C samples in different batches have been studied to verify the reproducibility of this cation-exchange approach (Supplementary Fig. 4c). To exclude the possibility of the presence of *N*-benzylquininium and Tb^3+^ cations residing on the surface of the crystallites, the cation-exchanged crystals were washed by *N*, *N*-dimethylformamide (DMF) six times over one day and the elutes were tested by CD and luminescent spectra. No detectable signal from CD spectra confirmed the absence of *N*-benzylquininium chloride of the elutes (Supplementary Fig. 5). Similarly, no detectable luminescence peak at 544 nm was observed on elutes from both Zn-MOF-Tb and Zn-MOF-C-Tb confirming that no free Tb^3+^ was released into the solution from the MOF system (Supplementary Fig. 6).

To explore the binding domains of *N*-benzylquininium cations and Tb^3+^ in Zn-MOF-C-Tb, we attempted to solve the structure of Zn-MOF-C-Tb by single crystal X-ray diffraction (SCXRD). However, the unambiguous determination of the location and orientation of the *N*-benzylquininium cations in the pore was unsuccessful due to its low occupancy and structure complexity. We also synthesized Zn-MOF-Tb by exchanging (CH_3_)_2_NH_2_^+^ by Tb^3+^ in the as-synthesized Zn-MOF to determine the positions of Tb^3+^ in the pore (Supplementary Fig. 1). Tb^3+^ ions have been located between two carboxyl groups on the linker stabilized by four oxygen donors (Supplementary Fig. 7). We used molecular modeling simulation at ultrafine level in Forcite module to study the positions of *N*-benzylquininium cations^36,37^. The Zn-MOF contains triangular and hexagonal channels with pore size of 0.8 nm and 3.0 nm. The simulation reveals that the most stable adsorption state is that half of the triangular channels were occupied by one *N*-benzylquininium cation and all of the hexagonal channels were occupied by five *N*-benzylquininium cations (Supplementary Fig. 8). The elemental analyses and thermal gravimetric analyses (TGA) (Supplementary Fig. 9) matched well the calculation based on the changes of the chemical formula. The porous nature of Zn-MOF-C has been studied by dye adsorption^38^. The different exchange rates of dyes of different molecular sizes (Supplementary Fig. 10) were in good agreements with the results of molecular modeling simulation.

### Fluorescence sensing

**Fig. 2:**
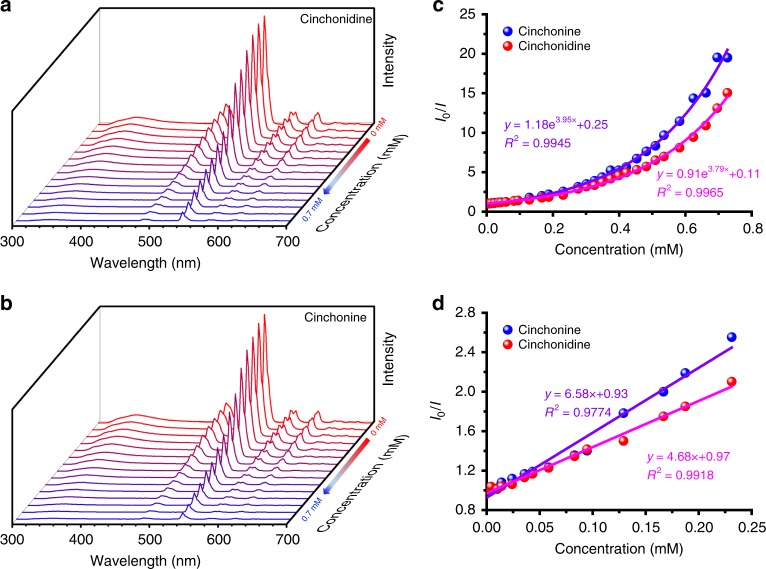
Emission spectra of Zn-MOF-C-Tb with Cinchonidine and Cinchonine. **a**, **b** Zn-MOF-C-Tb dispersed in DMF upon incremental addition of Cinchonidine and Cinchonine. **c**, **d** Fluorescence intensity changes at 544 nm and at lower concentrations. The solid lines are fitting results

The time-dependent fluorescence intensities of Zn-MOF-C-Tb and Zn-MOF-Tb in DMF solution were stable (Supplementary Figs. 11 and 12), enabling the fluorescent sensing measurements. To study the enantioselectivity of Zn-MOF-C-Tb, the epimers Cinchonidine and Cinchonine with multiple chiral centers were firstly studied as the target molecules (Supplementary Fig. 13a and b). The results showed that the quenching of the fluorescence at 544 nm by Cinchonine and Cinchonidine epimers followed the non-linear Stern-Volmer (S–V) behavior^39^ with different quenching rates between two epimers in the concentration range of 0–0.8 mmol L^−1^ (Fig. 2). The *K*_SV_ values are 4661 M^−1^ and 3449 M^−1^ based upon the fitting results of Fig. 2c by the empirical equation of *I*_0_/*I* = *a*·exp(*K*_SV_[C]) + *b* (or 6580 M^−1^ and 4680 M^−1^ based on the fitting results of Fig. 2d) for Cinchonine and Cinchonidine, respectively. The ratio of the *K*_SV_ values of Cinchonine to Cinchonidine is 1.4, indicating a preferential adsorption of Cinchonine by Zn-MOF-C-Tb, which has been further confirmed by the CD spectra where the mixture showed the signal of Cinchonidine after the addition of Zn-MOF-C-Tb (Supplementary Fig. 14). We further used Zn-MOF-Tb to clarify if the selective quenching behavior was induced by *N*-benzylquininium cations. The fluorescence of Zn-MOF-Tb can also be quenched by Cinchonine and Cinchonidine epimers but without any selectivity (Supplementary Fig. 15), clearly indicating that the epimeric and enantioselective recognition of Zn-MOF-C-Tb originates from its chiral centers in the pores.

**Fig. 3:**
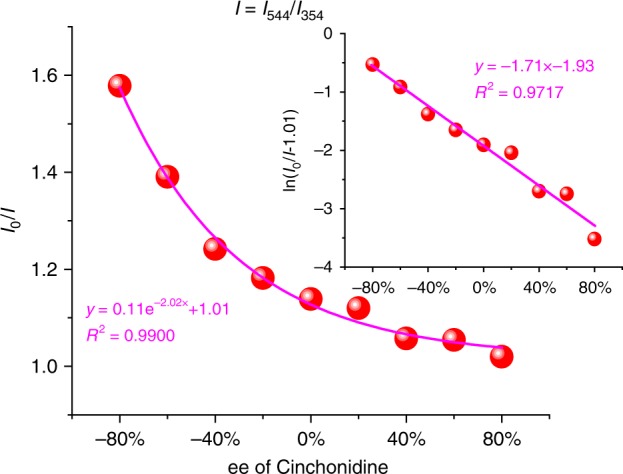
*ee* values with fluorescence intensities. Fluorescence intensities of Zn-MOF-C-Tb in DMF suspension versus *ee* values of Cinchonidine. Inset: the natural logarithm of the data. *I* in this figure is *I*_544_/*I*_354_

Based upon the different quenching behavior of Zn-MOF-C-Tb at 544 nm (Fig. 2) and 354 nm (Supplementary Fig. 16), the *ee* values can be obtained. The *ee* values of Cinchonidine were calculated by Eq. 1:

1 $$ee = \frac{{a - b}}{{a + b}} \times 100\%$$

where *a* and *b* are the concentrations of Cinchonidine and Cinchonine, respectively. The total concentration (*a* + *b*) can be calculated by *mV*/*M*, where *m* is the mass of the analytes, *V* is the volume of the MOF suspensions and *M* is the molecular mass of the analytes. In this case, *a* + *b* = 0.5 mmol L^−1^. The ratio of *I*_0_*/I* (*I* *=* *I*_544_/*I*_354_) decreased exponentially with the increasing *ee* values of Cinchonidine (Fig. 3 and Supplementary Fig. 17). A natural logarithm of the curve yields an equation of *y* = −1.71*x*−1.93 with excellent linearity between ln(*I*_0_/*I*–1.01) and *ee* values (R^2^ > 0.97).

**Fig. 4:**
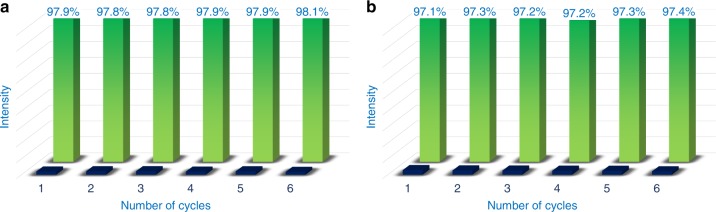
Recycling experiment and stability tests. The quenching ability of Zn-MOF-C-Tb dispersed in DMF in the presence of **a** Cinchonine and **b** Cinchonidine with six cycles (the green and blue bars represent the initial luminescence intensity and the intensity with 1 mmol L^−1^ analytes, respectively) at 544 nm. Numbers above the bars are the ratio of quantitative quenching

The reusability of this MOF material has been studied for six consecutive cycles. No change was observed for the luminescence intensities, the quenching efficiency or the epimeric recognition ability of Zn-MOF-C-Tb (Fig. 4).

### Underlying mechanism

In order to understand the underlying mechanism for observed epimeric recognition of Cinchonidine and Cinchonine within Zn-MOF-C-Tb, PXRD, the excitation spectra, the quantum yields, the luminescent lifetimes, solid-state NMR, liquid NMR and density functional theory (DFT) calculations were performed. PXRD (Supplementary Fig. 18) and recycling experiments (Fig. 4) revealed that there was no structural distortion of the local framework. The excitation spectra of suspensions of Zn-MOF-Tb, Zn-MOF-C-Tb in DMF with or without addition of Cinchonidine and Cinchonine at the strongest emission of 544 nm have been recorded (Supplementary Fig. 19). The excitation spectra of Zn-MOF-Tb with Cinchonidine or Cinchonine exhibit no notable difference, whereas those of Zn-MOF-C-Tb are different. This rules out the contingency for any excitation wavelength as each wavelength should efficiently excite the MOFs. The decline of the fluorescence intensities of Cinchonine was quicker than that of Cinchonidine in Zn-MOF-C-Tb indicating a greater influence of Cinchonine on the fluorescence of Zn-MOF-C-Tb than that of Cinchonidine. Also, the excitation wavelength was selected with the largest disparity (292 nm) in quenching efficiency for the fluorescence tests. The quantum yields (Supplementary Figs. 20, 21 and Table 1) and the luminescent lifetimes (Supplementary Figs. 22, 23 and Table 2) of Zn-MOF-C-Tb in DMF suspensions upon addition of Cinchonine were observed to decrease faster than those of Cinchonidine, confirming that Zn-MOF-C-Tb had better enantioselectivity luminescent sensing property for Cinchonine.

DFT calculations were carried out to study the interaction between the MOF and the analyte (Cinchonine and Cinchonidine) (Supplementary Fig. 24 and Table 3)^40^. The interaction energy of adsorbed molecules has been defined according to the formula:

2 $$\Delta E = E_{{\mathrm{M}} - {\mathrm{ch}}} - E_{\mathrm{M}} - E_{{\mathrm{ch}}}$$

where M stands for the MOF, *E*_M–ch_, *E*_M_ and *E*_ch_ are the energies of analytes-adsorbed MOF, MOF and pure analytes, respectively. For calculations of the potential interaction sites of Cinchonine and Cinchonidine with Zn-MOF-C, a 2 × 2 × 1 superlattice of Zn-MOF-C was created (including 4 hexagonal channels and 8 triangular channels). There are three possible alignment modes for Cinchonine and Cinchonidine epimers in Zn-MOF-C, namely center of hexagonal channel (Supplementary Fig. 24a and d), edge of hexagonal channel (Supplementary Fig. 24b and e) and center of triangular channels (Supplementary Fig. 24c and f).

Calculation results suggest that Zn-MOF-C shows stronger interactions with Cinchonine than Cinchonidine and the epimers prefer to reside at the edge of hexagonal channel and center of triangular channel, which can be attributed to electrostatic interaction, π···π stacking and hydrogen bond. Thus, the Zn-MOF and *N*-benzylquininium cations can attract Cinchonine synergistically via stronger host-guest interaction than that with Cinchonidine (Supplementary Table 3). Both the liquid and solid-state NMR studies further confirm the interaction between the analytes and the MOF host, consistent with the calculation result (Supplementary Figs. 25, 26).

**Fig. 5:**
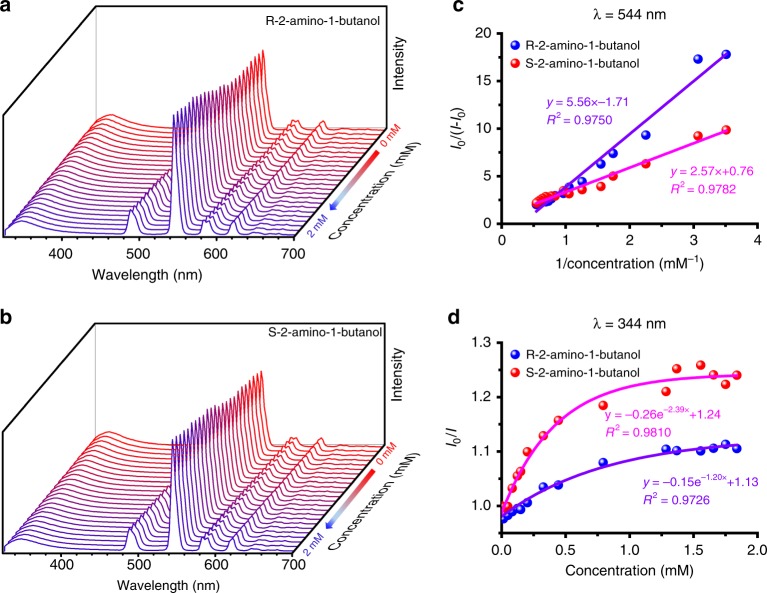
Emission spectra of Zn-MOF-C-Tb with R/S-2-amino-1-butanol. **a**, **b** Zn-MOF-C-Tb dispersed in DMF upon incremental addition of R-2-amino-1-butanol and S-2-amino-1-butanol. **c**, **d** Fluorescence intensity changes of Zn-MOF-C-Tb at 544 nm and 344 nm. The solid lines are fitting results

To demonstrate the general applicability of the bifunctional MOF, the sensing properties of a range of enantiomers of *N*-benzylcinchoninium chloride/*N*-benzylcinchonidinium chloride (Supplementary Figs. 27 to 36, Tables 4, 5), R-2-amino-1-butanol/S-2-amino-1-butanol (Fig. 5, Supplementary Figs. 37 to 44, Tables 6, 7) and R-2-amino-1-propanol/S-2-amino-1-propanol (Supplementary Figs. 45 to 53, Tables 8, 9) were investigated. *N*-benzylcinchoninium chloride and *N*-benzylcinchonidinium chloride are also drugs and asymmetric catalytic agent^41,42^. Chiral amino alcohols are important compounds in organic synthesis and precursors for drugs^43,44^. Zn-MOF-C-Tb showed an excellent enantioselectivity in all cases. The quenching of florescence of *N*-benzylcinchoninium chloride, S-2-amino-1-butanol and S-2-amino-1-propanol were faster than their corresponding enantiomers, demonstrating the broader application of this bifunctional MOF system. Interference experiments were also carried out by varying the scan slit size, the excitation wavelength and the MOF concentration (Supplementary Fig. 54). The relative standard deviation for the ratio of *I*_544_*/I*_354_ was significantly lower than that of using the *I*_544_ emission alone, further demonstrating the advantage of minimized system errors for the ratiometric sensing approach.

## Discussion

Compared with other materials with chiral structures for enantioselective recognition (Supplementary Table 10), Zn-MOF-C-Tb has shown both excellent sensitivity and high enantioselectivity. This bifunctional MOF has three key advantages. (i) It is a dual-center sensor with emissions from both ligands and metal centers in the pore, and thus can be used for ratiometric sensing with enhanced recognition ability and accurate determination of *ee* values of mixtures of stereoisomers (epimers and enantiomers). (ii) The cost to synthesize this MOF with multiple chiral centers is notably cheaper than state-of-the-art systems based upon complex, chiral organic linkers. This is because the chiral center (*N*-benzylquininium chloride) is commercialized and very small amount of the chiral reagent was used to induce the enantioselective recognition property. (iii) This method can be potentially extended to other combinations of anionic MOFs and other chiral cations, i.e., to achieve tailored-to-property design.

In summary, we report a novel strategy to construct bifunctional (chiral and luminescent) MOFs for enantioselective fluorescence recognition and quantitative determination of *ee* values for enantiomers. The competition for the absorption of the enantiomers to the post-modified pore chirality is responsible for the recognition property. Based upon the methods developed here, anionic MOFs can be generally modified to be used for enantioselective fluorescence recognition, paving a new pathway for the design and development of new functional sensors of organic chiral molecules.

## Methods

### Materials and methods

All reagents were commercially available and used without further purification. H_3_TATAB was synthetized according to literature^45^. ^1^H NMR spectra for Zn-MOF and Zn-MOF-C were recorded on a Mercury Vx-300 MHz NMR spectrometer. ^1^H NMR spectra for Zn-MOF-C-Tb and the analytes were recorded on a Bruker Ascend 500 NMR spectrometer. ^13^C NMR spectra for the analytes and the MOF were measured on a Bruker Avance NEO 400 MHz wide bore solid-state NMR spectrometer. PXRD measurements were performed using a Rigaku D/Max-2500 x-ray diffractometer with Cu-Kα radiation. TGA were obtained under nitrogen atmosphere on NETZSCH TG209 Setaram from 40 °C to 800 °C. SCXRD measurement for Zn-MOF-Tb was tested at Diamond Light Source Beamline I19. The structure for Zn-MOF-Tb was solved by a standard direct method and refined with anisotropic thermal parameters by the least-squares method on *F*^2^ using the SHELXTL software package^46^. The CCDC number is 1909508. CD spectra were taken by ChirascanPlus at Diamond Light Source Beamline B23. Liquid CD spectra were measured on JASCO J-715 circular dichroism. Elemental analyses for C, H and N were carried out using a Vario EL cube elemental analyzer. Ion chromatography was performed by DIONEX ICS-1100. Inductively coupled plasma (ICP) analysis were conducted using a Thermo IRIS Advantage instrument after degradation of the samples in aqua regia. Energy dispersive spectroscopy (EDS) were tested by Hitachi SU3500 with Bruker energy spectrometer. Luminescence spectra, luminescence lifetime and external quantum yield were recorded on an Edinburgh FS5 fluorescence spectrophotometer equipped with Xenon lamp, pulsed flash lamps and an integrating sphere. Ultraviolet-Visible (UV-Vis) absorption spectra were measured with Agilent Cary 100 UV-Vis Spectrometer. Molecular simulation was calculated by Forcite function and Dmol^3^ of Materials Studio 6.1. High-performance liquid chromatography was tested at Agilent 1260 Infinity by AS-H chromatographic column using hexane/iPrOH/Et_2_N = 90:10:0.1.

### Synthesis of Zn-MOF

Zn-MOF was prepared via a modified method reported previously with nitric acid instead of fluoroboric acid^33^. Zn(NO_3_)_2_·6H_2_O (119.4 mg, 0.4 mmol), adenine (ad, 27 mg, 0.2 mmol), H_3_TATAB (97.3 mg, 0.2 mmol), DMF (13 mL) and nitric acid (68%, 150 μL) were added into a 23 mL Teflon-lined stainless steel vessel. Then the sealed vessel was placed into an oven at 130 °C for 48 h. Elemental analysis (%) calculated for C_48_H_73_N_17.5_O_16.25_Zn_2_ ([(CH_3_)_2_NH_2_]_0.5_[Zn_2_(ad)(TATAB)O_0.25_](DMF)_6_(H_2_O)_4_): C 44.83, H 5.72, N 19.06; found: C 44.67, H 5.08, N 19.04.

### Synthesis of Zn-MOF-C

Fifty milligram Zn-MOF was added to the solution of *N*-benzylquininium chloride (45.1 mg, 0.1 mmol) in 10 mL DMF. Seven days later, the dimethylamine ions were exchanged completely by *N*-benzylquininium ions according to the ^1^H NMR patterns. The product was collected by filtering and soaked in DMF overnight. Then it was washed with large amounts of fresh DMF until the eluate exhibited no evident Cotton effects. No chloride was found in Zn-MOF-C from ion chromatography or EDS. Elemental analysis (%) calculated for C_60_H_84_N_18_O_17.25_Zn_2_ ((*N*-benzylquininium)_0.5_[Zn_2_(ad)(TATAB)O_0.25_](DMF)_6_(H_2_O)_4_): C 49.21, H 5.78, N 17.22; found: C 49.18, H 5.03, N 17.16.

### Synthesis of Zn-MOF-Tb

A solution of TbCl_3_·6H_2_O (18.7 mg, 0.05 mmol) in 10 mL DMF was added into 20 mL glasses (5 mmol L^−1^). 50 mg Zn-MOF was added for 30 min. The product was collected by filtering and soaked in DMF overnight. Then it was washed with large amounts of fresh DMF until the eluate exhibited no obvious luminescent signals. Elemental analysis (%) calculated for C_47.64_H_71.80_N_17.32_O_16.37_Zn_2_Tb_0.06_ ([(CH_3_)_2_NH_2_]_0.32_[Tb(H_2_O)_2_]_0.06_[Zn_2_(ad)(TATAB)O_0.25_](DMF)_6_(H_2_O)_4_): C 44.37, H 5.61, N 18.81, Tb 0.74; found: C 43.91, H 5.22, N 18.62. The content of Tb in Zn-MOF-Tb is 0.70% according to the ICP analysis and 1.03% by EDS.

### Synthesis of Zn-MOF-C-Tb

Zn-MOF-C-Tb was synthesized in the similar way as Zn-MOF-Tb, except that Zn-MOF-C was used instead of Zn-MOF. Elemental analysis (%) calculated for C_53.76_H_77.12_N_17.52_O_16.93_Zn_2_Tb_0.08_ ((*N*-benzylquininium)_0.26_[Tb(H_2_O)_2_]_0.08_[Zn_2_(ad)(TATAB)O_0.25_](DMF)_6_(H_2_O)_4_): C 46.68, H 5.61, N 17.74, Tb 0.92; found: C 46.18, H 4.97, N 17.84. The content of Tb in Zn-MOF-C-Tb is 1.42% according to the ICP analysis and 1.95% by EDS.

### NMR tests

Zn-MOF-C and Zn-MOF-C-Tb were multiple time washed with fresh DMF and then soaked in ethanol to exchange DMF overnight in order to avoid the infections of the decompose of DMF. The samples were collected by filtering. 3 mg of each sample was dissolved in 0.6 mL DMSO-*d*_6_ with 5 μL DCl. Parallel experiments in other glasses were carried out to clarify the phenomenon with other glasses’ samples. 3 mg *N*-benzylquininium chloride was dissolved in 0.6 mL DMSO-*d*_6_ for comparison. For liquid ^1^H NMR spectra, DMF solutions of raw materials (10 mM Cinchonine/Cinchonidine, adenine-ligand-1, H_3_TATAB-ligand-2, *N*-benzylquininium chloride) and the mixtures of ligands/chiral center/La^3+^ and epimers (10 mM adenine and Cinchonine/Cinchonidine, H_3_TATAB and Cinchonine/Cinchonidine, *N*-benzylquininium chloride and Cinchonine/Cinchonidine, LaCl_3_ and Cinchonine/Cinchonidine) were tested. La^3+^ was used to simplify the paramagnetic property of Tb^3+^. Each 50 μL of these solutions were mixed with 500 μL *d*_6_-DMSO. For solid-state ^13^C NMR patterns of the analytes and the MOF, 30 mg Zn-MOF-C was dispersed in 5 mL DMF with 2 mg Cinchonine for 7 days to construct the sample of Zn-MOF-C with Cinchonine.

### CD measurements

Solid-state CD spectra of Zn-MOF-C were carried out with 1 mg Zn-MOF-C and 100 mg KBr using a Chirascan Plus CD spectrophotometer (Applied Photophysics, UK). The samples for liquid-state CD measurements were diluted accordingly in order not to exceed 600 V of the HT value. To make sure no *N*-benzylquininium chloride left in Zn-MOF-C, a solution of *N*-benzylquininium chloride (45.1 mg, 0.1 mmol) in 10 mL DMF was diluted 20 times with DMF. The solution of *N*-benzylquininium chloride exhibited negative Cotton effects in the wavelength range 290–360 nm whilst the final eluate with DMF did not indicate its absence. For the adsorption tests, 1 mL of the 20 mg (0.068 mmol) Cinchonine/Cinchonidine, which were dissolved into 50 mL DMF were diluted 10 times with DMF for the measurement. And 10 mL of each was mixed up, 30 mg Zn-MOF-C-Tb were added. The intensities changed with time. Similar conditions were tested for *N*-benzylcinchoninium chloride/*N*-benzylcinchonidinium chloride, 2-amino-1-butanol and 2-amino-1-propanol.

### Photoluminescence experiments

Zn-MOF-Tb and Zn-MOF-C-Tb were grinded into fine powder before use. The samples for photoluminescence experiments were dispersed in DMF by ultrasound for 30 min with the concentration of 1 mg mL^−1^. The concentrations for Cinchonine/Cinchonidine (the purities of Cinchonine and Cinchonidine have been confirmed by high-performance liquid chromatography, Supplementary Fig. 55) and *N*-benzylcinchoninium chloride/*N*-benzylcinchonidinium chloride were 2 mg/5 mL in DMF and 2 μL/5 mL for 2-amino-1-butanol and 2-amino-1-propanol. The spectra of the detections of Cinchonine/Cinchonidine and *N*-benzylcinchoninium chloride/*N*-benzylcinchonidinium chloride excited at 292 nm were recorded from 300 to 700 nm and 314 nm for 2-amino-1-butanol and 2-amino-1-propanol recorded from 318 to 700 nm. Each spectrum was recorded after the analytes were added and sonicated for 1 min and the intensity was stabilized (Supplementary Fig. 56). According to the lifetime (Supplementary Tables 2, 5) of the peaks at 544 nm, the quenching processes of Tb^3+^ are dynamic by Cinchonine and *N*-benzylcinchoninium chloride and static by 2-amino-1-butanol and 2-amino-1-propanol, respectively (Supplementary Tables 7, 9). The quenching processes by the ligand are all dynamic. Tb^3+^ can interact with the -OH group of these amino alcohols, thus improving the energy transfer efficiency from the ligand to Tb^3+^. 2-amino-1-butanol and 2-amino-1-propanol are better antenna than water for Tb^3+^ (Supplementary Fig. 57).

### DFT calculations of the structure and the energy

For Zn-MOF-C, we selected one cell which contains a layer of one hexagon channel and two triangle channels for the calculation (Supplementary Fig. 8). Six *N*-benzylquininium cations exist in one cell according to the molecular formula determined by elemental analysis and TGA. One triangle channel contains only one *N*-benzylquininium cation while six *N*-benzylquininium cations can exist in the large pore at most. Thus, there are totally three possible structures namely [6 + 0], [5 + 1], and [4 + 2]. Molecular dynamic (MD) simulation reveals that the most stable pattern is the second one with 50% small channels occupied and five ions in large channels. According to the Boltzmann distribution law, such a pattern takes the majority. To investigate the mechanism of selective adsorption of Zn-MOF-C for Cinchonine and Cinchonidine, DFT calculation was performed using the DMol^3^ program of Materials Studio. To describe the approximation of exchange-correction energy, gradient-corrected functional developed by Perdew et al (GGA-PBE) was chosen for geometry optimization^40^. The weak van der Waals interaction was considered by introduction of semi-empirical Grimme dispersion correction method. Besides, double numerical plus polarization basis set DNP 4.4 in DMol^3^ was applied and all core electrons of atoms were taken into consideration. The convergence criterions of total energy, force, and displacement were set as 10^−6^ Ha, 0.002 Ha Å^−1^, and 0.005 Å. HOMO-LUMO energy levels and energy variation were calculated with the DMol^3^ program package by Material Studio 6.1. According to the results, the LUMO of *N*-benzylcinchoninium chloride is obviously lower than that of the H_3_TATAB and adenine (Supplementary Table 11). This indicates the presence of the PET (photo induced electron transfer) process for the luminescent quenching^5,19^. From the UV-vis absorption spectroscopy (Supplementary Fig. 58), the competitions for the absorption of irradiated light are also attributed to the quenching^19^.

### Porosity test for Zn-MOF and Zn-MOF-C

To test the porosity of Zn-MOF and Zn-MOF-C in solution, dye adsorption experiments were performed. These MOFs have good ability for the adsorption of methyl blue, crystal violet and rhodamine 6 G. Dye-uptake were monitored by UV-Vis absorption spectra. Triplicate 12.9 mg Zn-MOF (contains 0.01 mmol ligand according to the formula) and 14.6 mg Zn-MOF-C (contains 0.01 mmol ligand according to the formula) were transferred separately into a DMF solution (4 mL) of methyl blue (2.5 × 10^−5^ mol L^−1^), crystal violet (2 × 10^−4^ mol L^−1^), and rhodamine 6 G (2 × 10^−5^ mol L^−1^), respectively. The extraction of the upper clear solution was used for UV-Vis absorption measurement after 30, 60, and 90 min.

## Supplementary information

Supplementary information

## Data Availability

The data that support the findings of this study are available from the corresponding author.

## Supplementary information

**Supplementary information** is avaliable for this paper at 10.1038/s41467-019-13090-9.
