# Supplementary material for: Cation-induced chirality in a bifunctional metal-organic framework for quantitative enantioselective recognition
Source: Nat Commun. 2019 Nov 11;10:5117. doi: 10.1038/s41467-019-13090-9 (PMC6848213; doi:10.1038/s41467-019-13090-9)
Supplement: Supplementary file 1 — Supplementary information [file 41467_2019_13090_MOESM1_ESM.pdf]

Cation-induced chirality in a bifunctional metal-organic framework for quantitative enantioselective recognition

Han et al.

Supplementary Figures and Tables

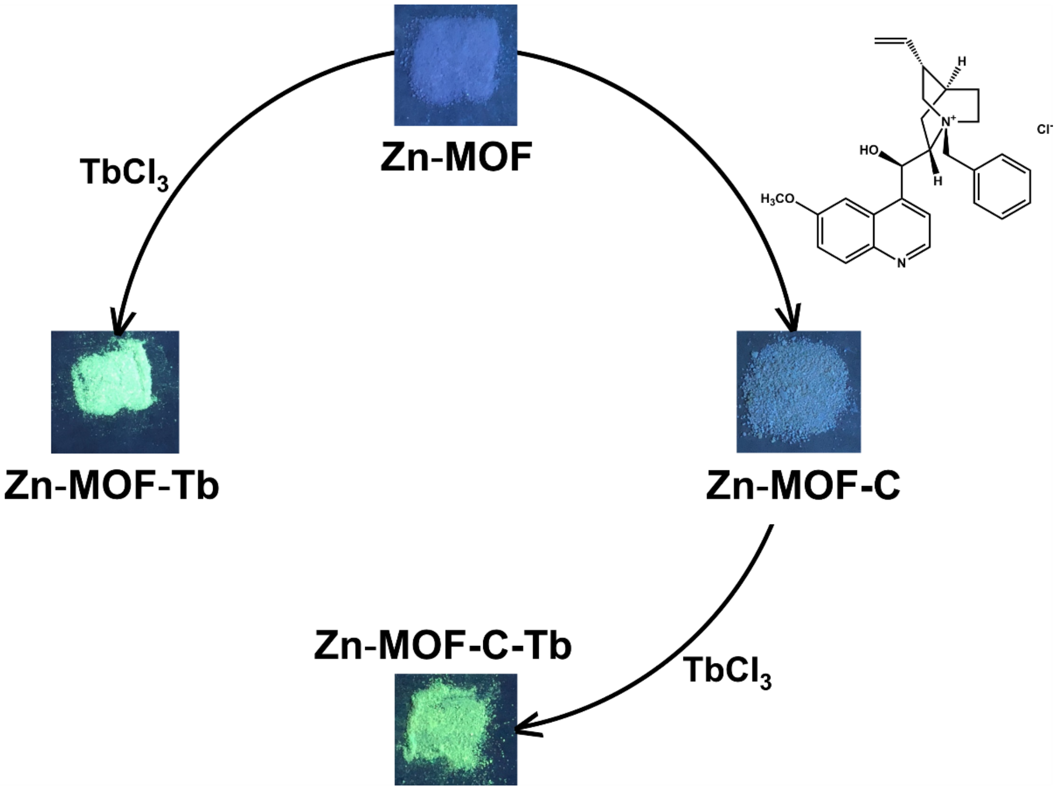

Supplementary Figure 1. Synthesis methods. Synthesis of Zn-MOF-Tb, Zn-MOF-C and Zn-MOF-C-Tb.

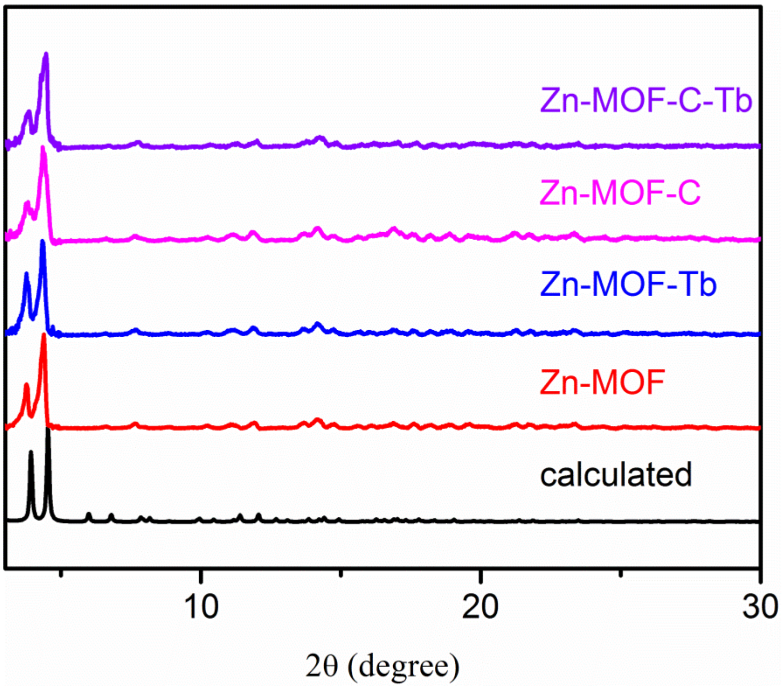

Supplementary Figure 2. PXRD patterns. PXRD patterns for calculated Zn-MOF, as-synthesized Zn-MOF, Zn-MOF-Tb, Zn-MOF-C and Zn-MOF-C-Tb.

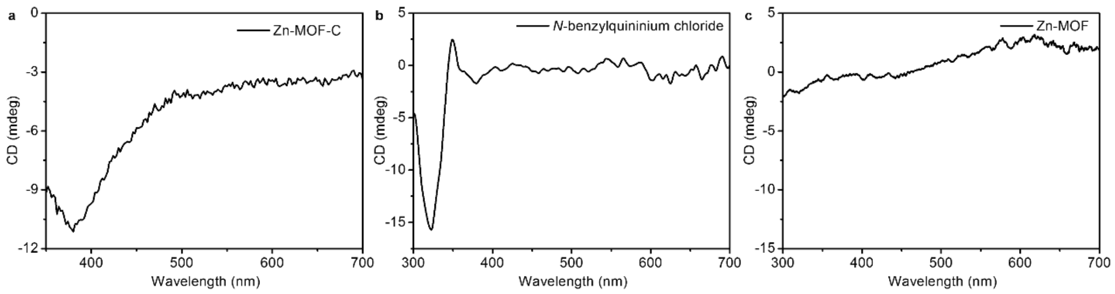

Supplementary Figure 3. Solid-state CD spectra. (**a**) Zn-MOF-C (**b**) *N*-benzylquininium chloride and (**c**) Zn-MOF.

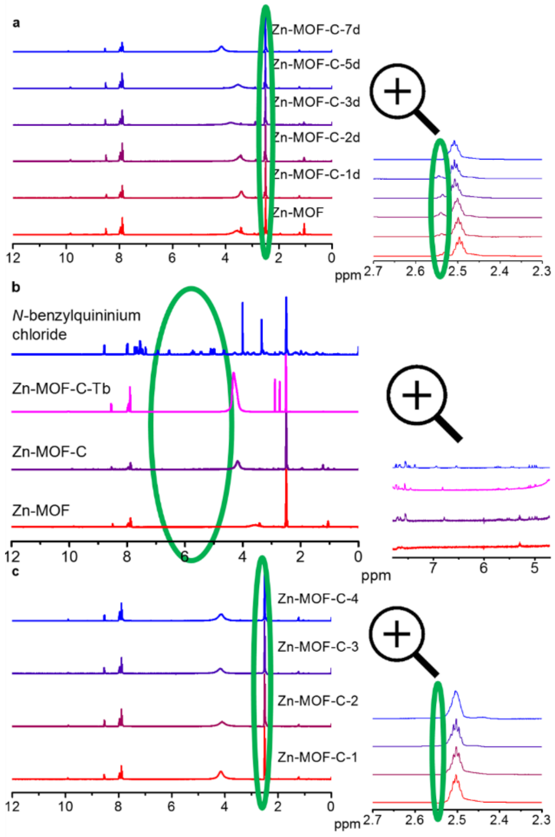

Supplementary Figure 4. ^{1}H NMR spectra. (**a**) Zn-MOF soaked in *N*-benzylquininium chloride from the initial status to 7 days. The peak of methyl hydrogen of dimethylamine cations gradually weakens with time. (**b**) Zn-MOF, Zn-MOF-C, Zn-MOF-C-Tb and *N*-benzylquininium chloride (the intensities for Zn-MOF, Zn-MOF-C and Zn-MOF-C-Tb at the right picture have been magnified by about 10 times). Most of the peaks of both Zn-MOF and *N*-benzylquininium chloride can be seen in Zn-MOF-C and Zn-MOF-C-Tb. (**c**) Zn-MOF soaked in *N*-benzylquininium chloride at the 7 day for parallel experiments. The peaks at 2.54 ppm corresponding to methyl disappeared completely.

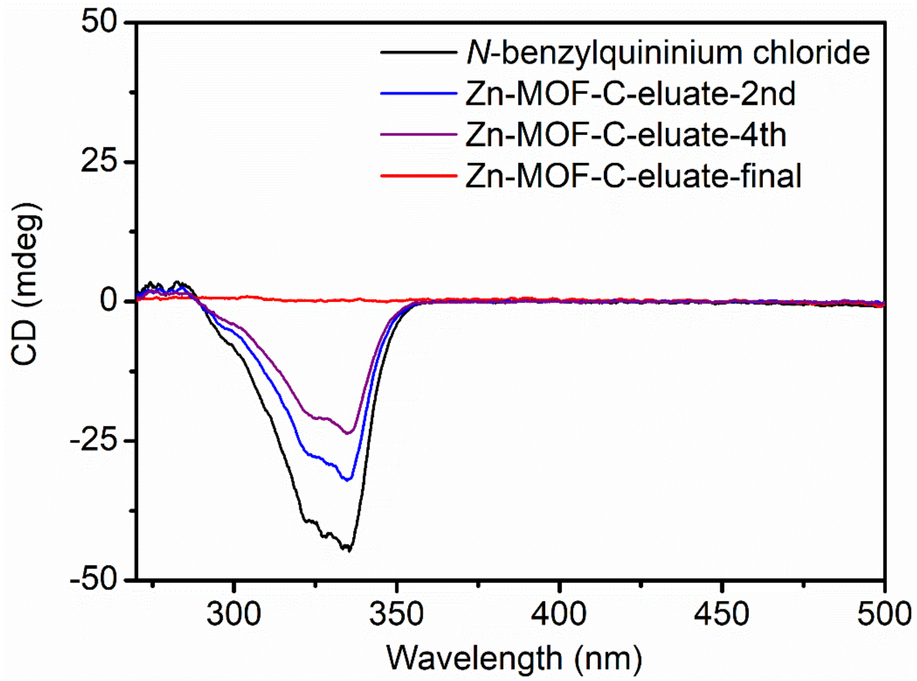

Supplementary Figure 5. Liquid-state CD spectra. CD spectra of 20 times dilution of the DMF solution of 10 mmol L^{-1} *N*-benzylquininium chloride and the eluates of the Zn-MOF-C.

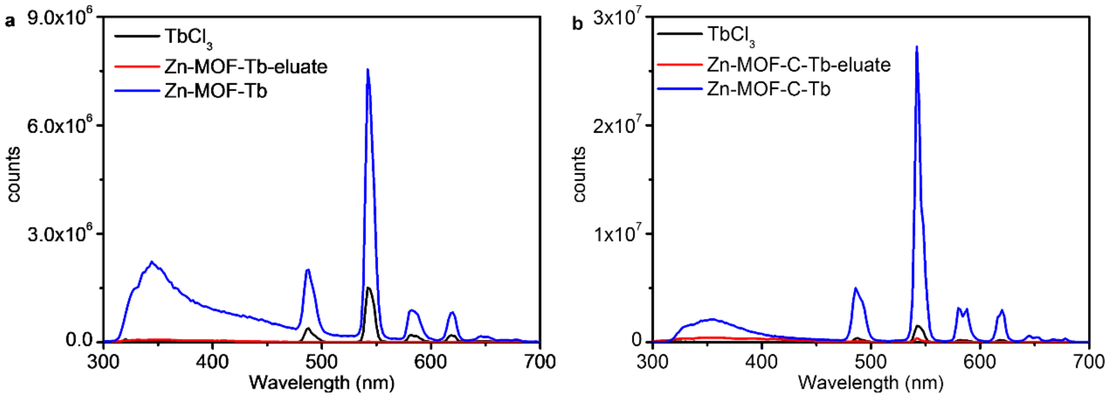

Supplementary Figure 6. Fluorescence emission spectra excited at 292 nm. (**a**) 5 mmol L^{-1} $TbCl_3$, the eluates of Zn-MOF-Tb and Zn-MOF-Tb suspensions in DMF and (**b**) 5 mmol L^{-1} $TbCl_3$, the eluate of Zn-MOF-C-Tb and Zn-MOF-C-Tb suspensions in DMF.

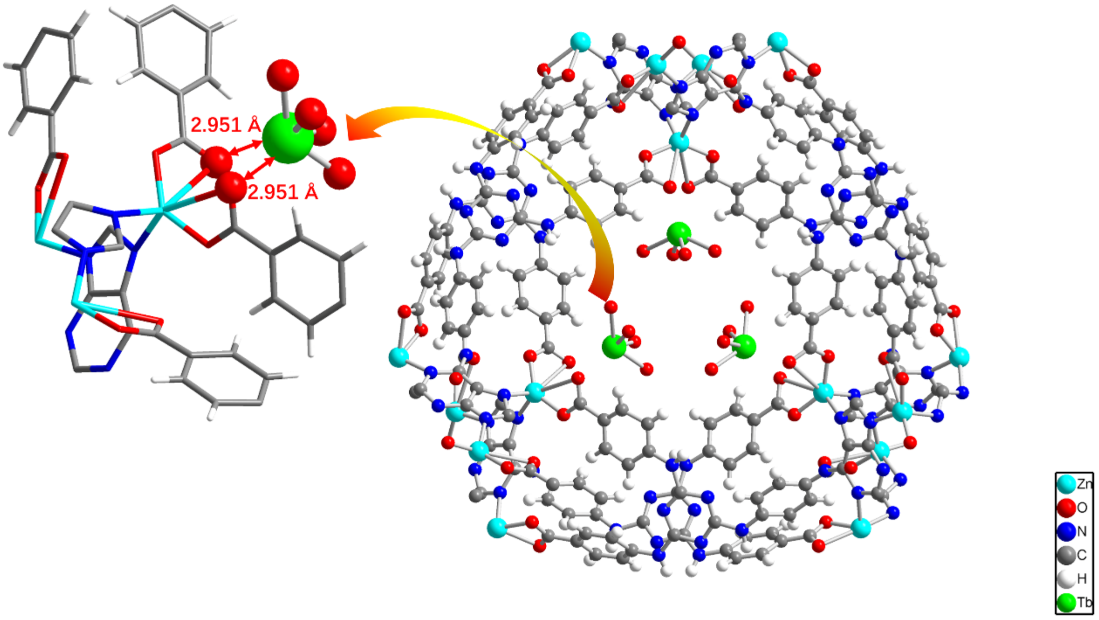

Supplementary Figure 7. The position for Tb cations in Zn-MOF-Tb. There is a new biggest Q peak (electron density about 6.6) in Zn-MOF-Tb beyond the framework compared with Zn-MOF. We considered it as about 0.25 Tb with several highly disordered water.

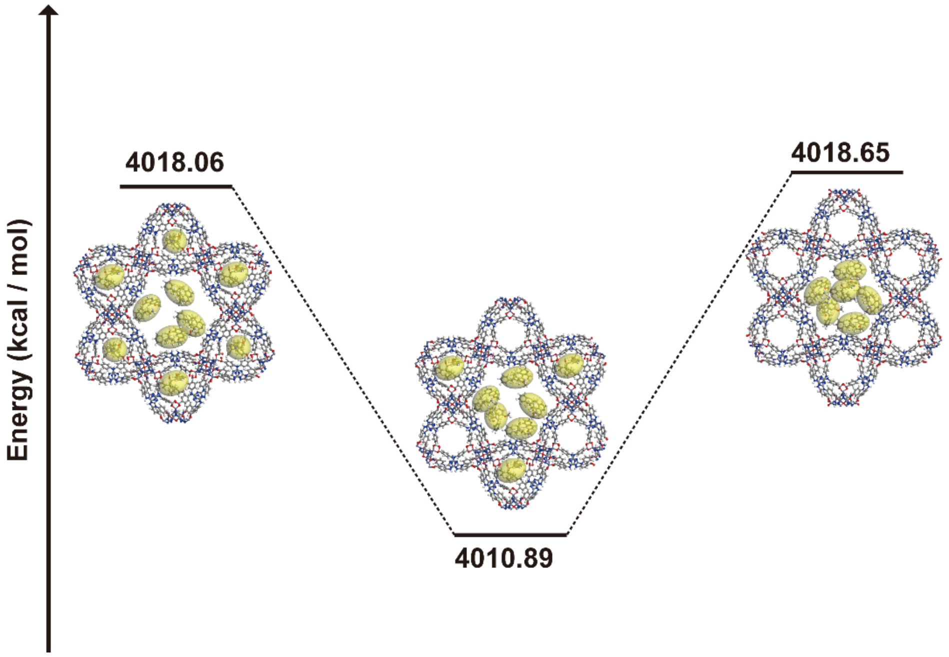

Supplementary Figure 8. Three possible distribution patterns of *N*-benzylquininium cations in Zn-MOF-C. From left to right, the occupations of triangle channels of Zn-MOF are 100%, 50%, and 0 and the number of *N*-benzylquininium cations in large pores are 4, 5, and 6, respectively. According to Boltzmann distribution law, the middle one is the major.

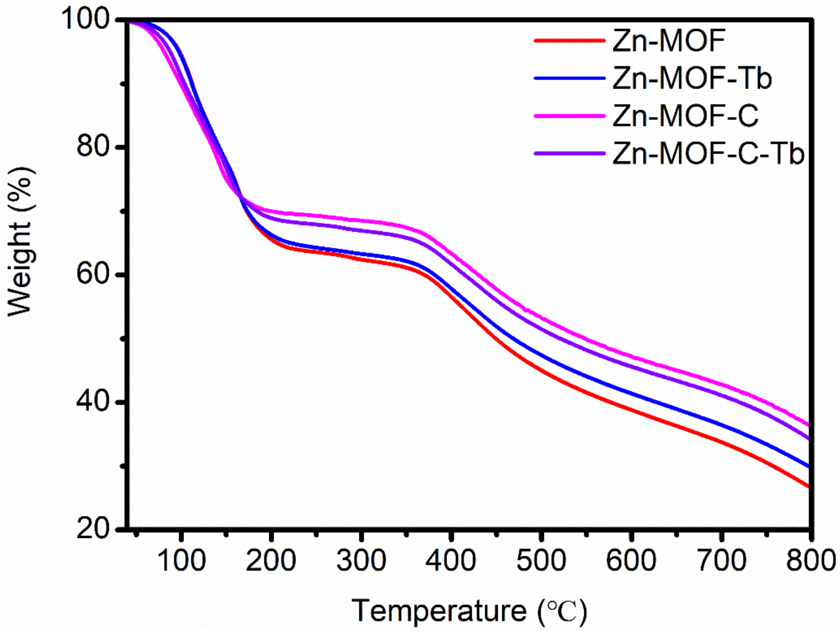

Supplementary Figure 9. TGA patterns. TGA patterns of Zn-MOF, Zn-MOF-Tb, Zn-MOF-C and Zn-MOF-C-Tb.

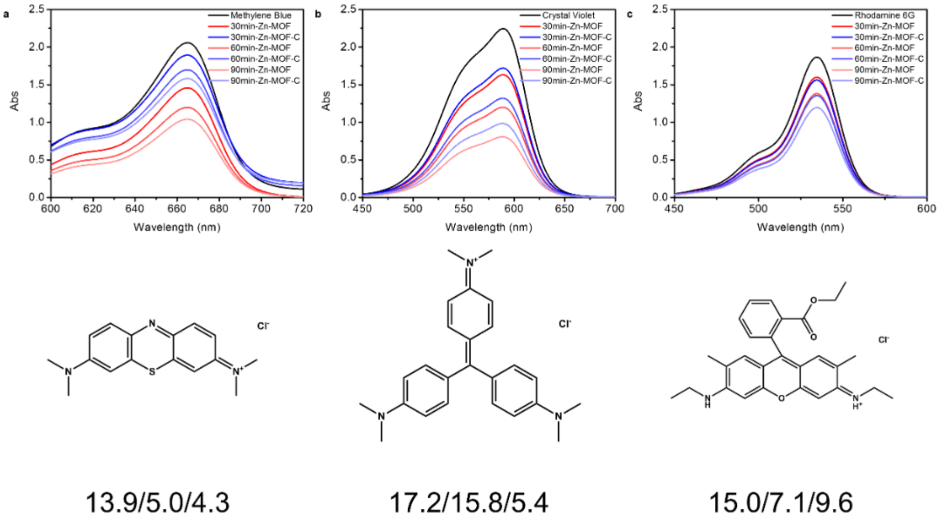

Supplementary Figure 10. UV-vis spectra of the solutions in the dye adsorption processes by Zn-MOF and Zn-MOF-C. (**a**) Methylene blue (**b**) Crystal violet (**c**) Rhodamine 6G. For crystal violet, the huge shape of this dye makes it only go through the large channel. The decrease of the adsorption of crystal violet reveals some chiral molecules are in the large channel. For rhodamine 6G, the size is much smaller than crystal violet. The large channel of Zn-MOF-C can also accommodate this dye. In the case of methyl blue, which can both get into the large channel and the small channel, the decline of the adsorption ability announces *N*-benzylquininium cations are in both the large and small channels.

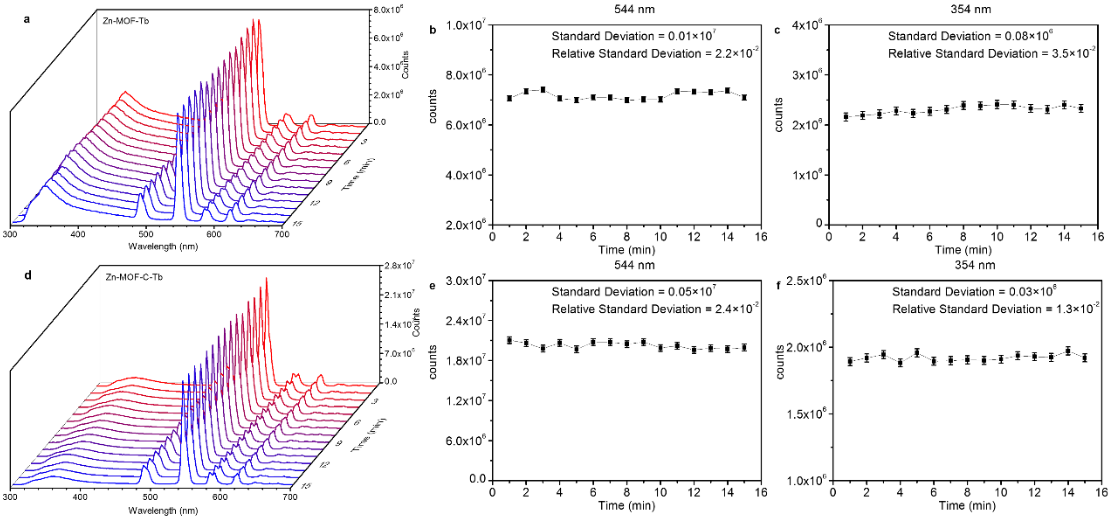

Supplementary Figure 11. Fluorescence emission spectra excited at 292 nm. (**a**) Zn-MOF-Tb suspensions in DMF for 15 minutes and the fluorescence intensity of Zn-MOF-Tb at (**b**) 544 nm, (**c**) 354 nm. (**d**) Zn-MOF-C-Tb suspensions in DMF for 15 minutes and the fluorescence intensity of Zn-MOF-C-Tb at (**e**) 544 nm, (**f**) 354 nm.

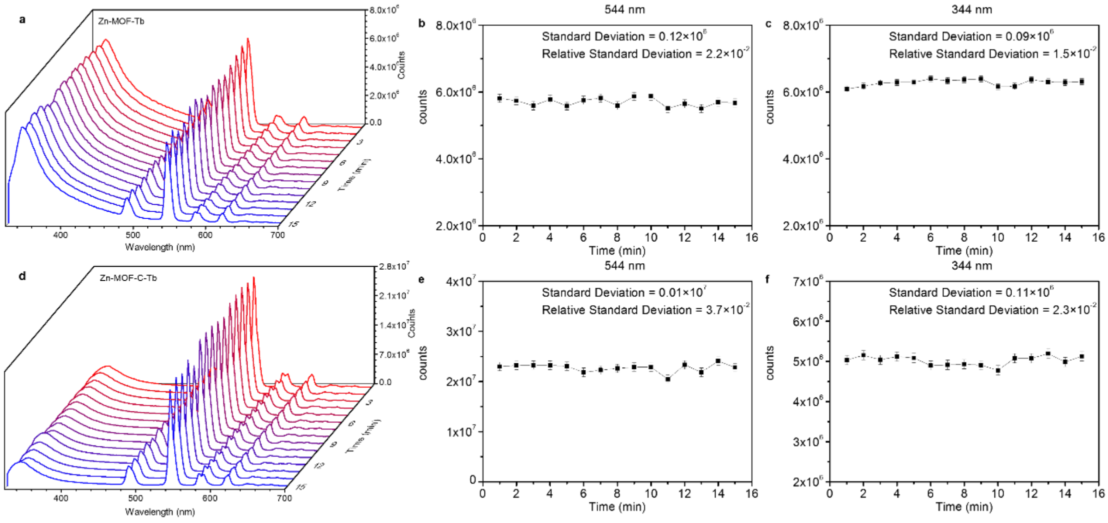

Supplementary Figure 12. Fluorescence emission spectra excited at 314 nm. (**a**) Zn-MOF-Tb suspensions in DMF for 15 minutes and the fluorescence intensity of Zn-MOF-Tb at (**b**) 544 nm, (**c**) 344 nm. (**d**) Zn-MOF-C-Tb suspensions in DMF for 15 minutes and the fluorescence intensity of Zn-MOF-C-Tb at (**e**) 544 nm, (**f**) 344 nm.

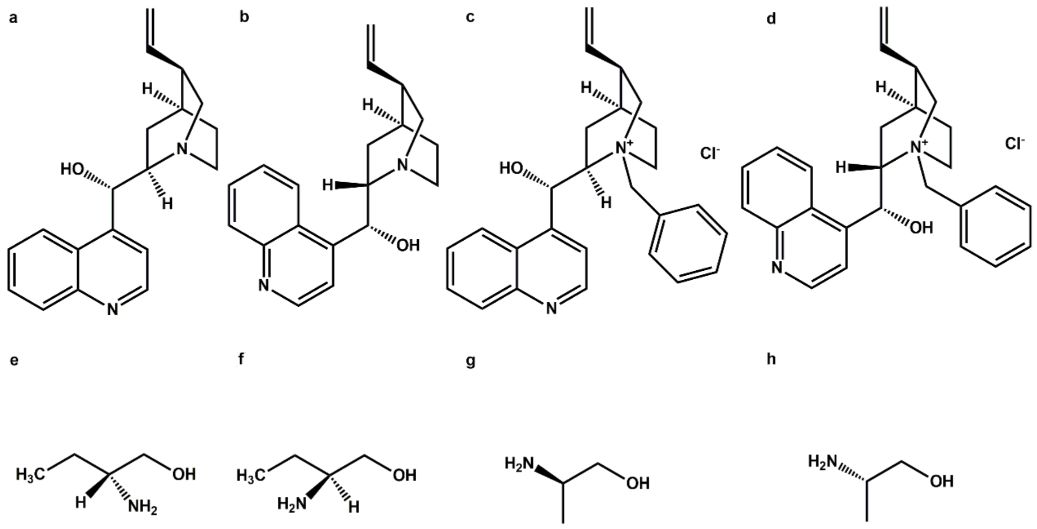

Supplementary Figure 13. The structures of the analytes. (**a**) Cinchonine and (**b**) Cinchonidine, (**c**) *N*-benzylcinchoninium chloride and (**d**) *N*-benzylcinchonidinium chloride, (**e**) R-2-amino-1-butanol and (**f**) S-2-amino-1-butanol, (**g**) R-2-amino-1-propanol and (**h**) S-2-amino-1-propanol.

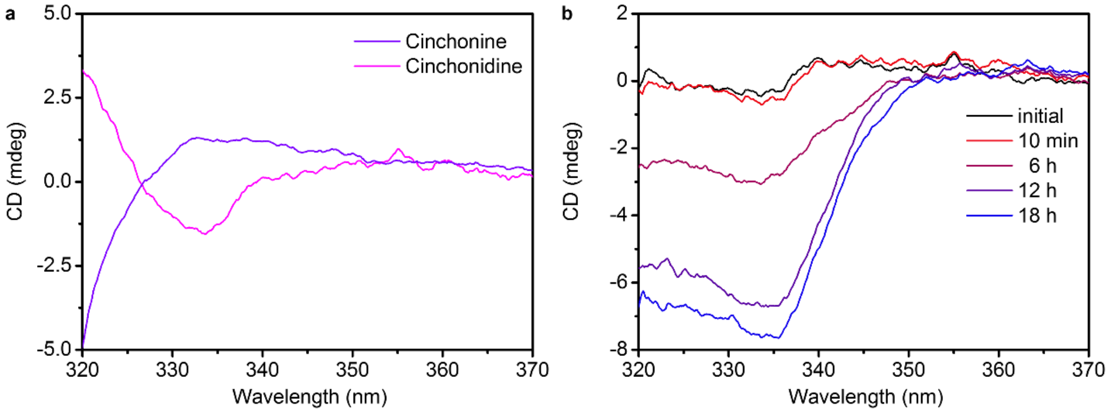

Supplementary Figure 14. Liquid-state CD spectra. (**a**) 10 times dilution of the DMF solution of 1.36 mmol L^{-1} Cinchonine and Cinchonidine (2 mg-5 mL) and (**b**) the intensity changes of the equal proportion of the mixture with the addition of Zn-MOF-C-Tb.

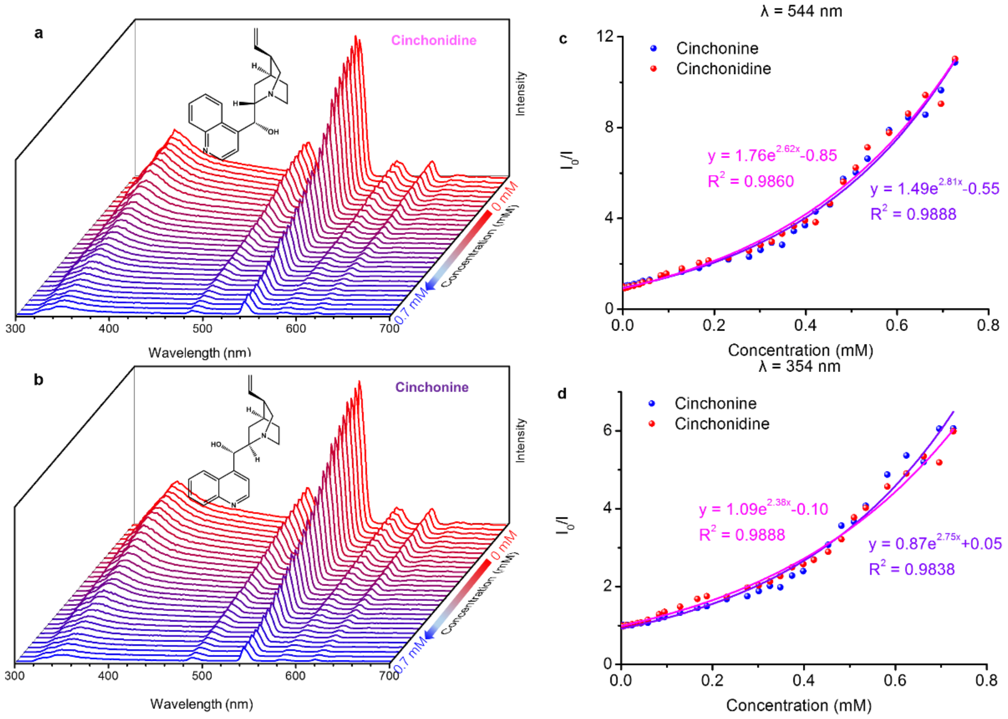

Supplementary Figure 15. Fluorescence emission spectra excited at 292 nm. Zn-MOF-Tb dispersed in DMF upon incremental addition of (**a**) Cinchonidine and (**b**) Cinchonine. Fluorescence intensity changes of Zn-MOF-Tb at (**c**) 544 nm and (**d**) 354 nm.

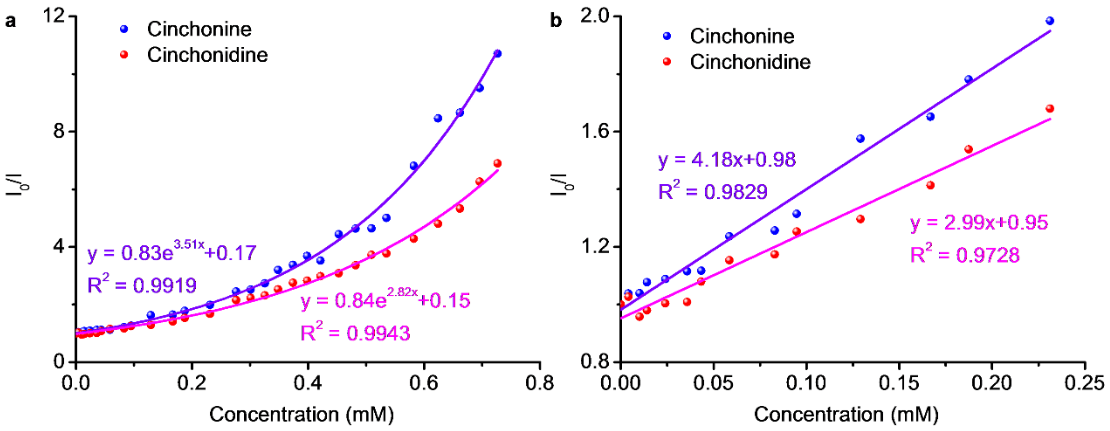

Supplementary Figure 16. Fluorescence intensity changes. Fluorescence intensity changes of Zn-MOF-C-Tb at (**a**) 354 nm (**b**) 354 nm at lower concentrations (0-0.25 mmol L^{-1}).

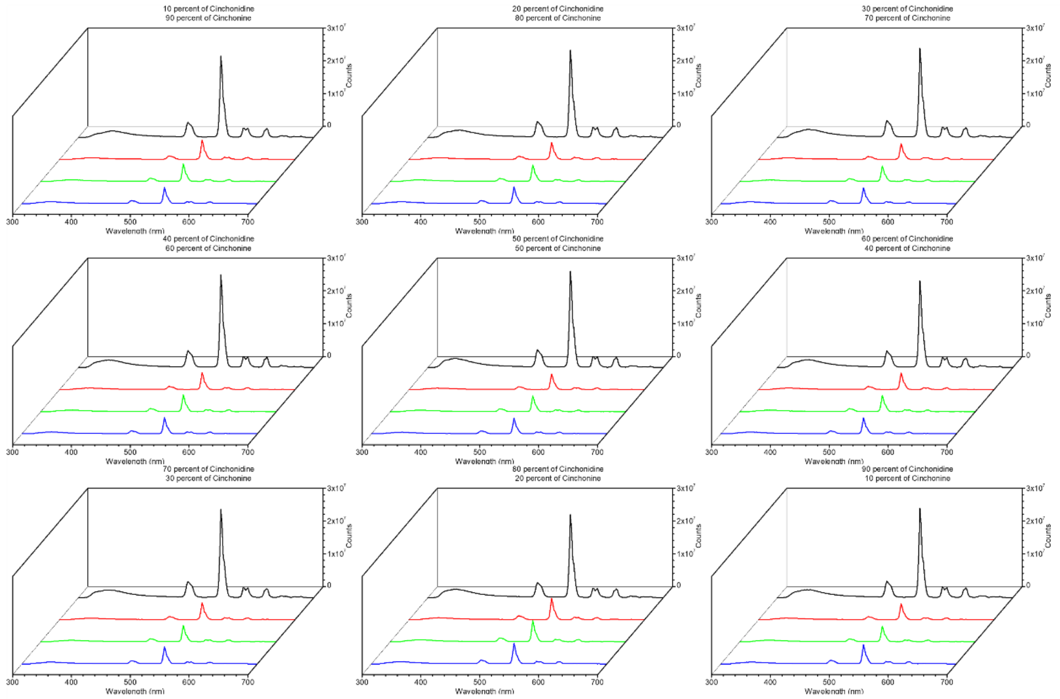

Supplementary Figure 17. Fluorescence intensity changes of Zn-MOF-C-Tb toward addition of a mixture of different proportions of Cinchonidine and Cinchonine. Black lines represent the original Zn-MOF-C-Tb while others stand for Zn-MOF-C-Tb with the mixtures of different *ee* values for three times.

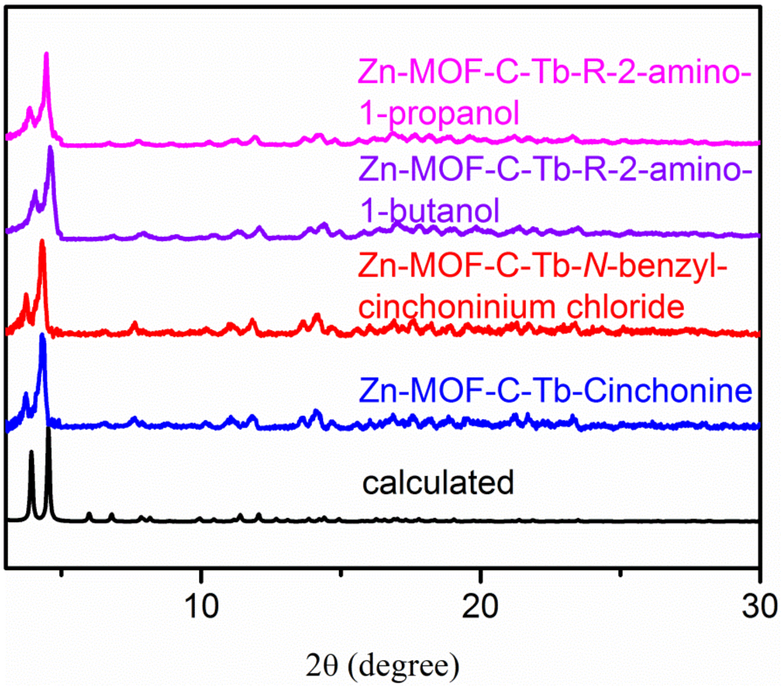

Supplementary Figure 18. PXRD patterns. PXRD patterns of Zn-MOF-C-Tb after soaking in additions for about 1 hour.

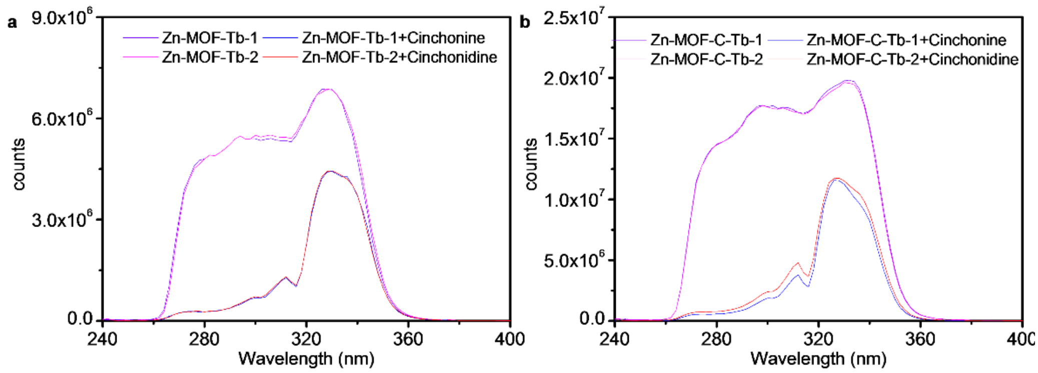

Supplementary Figure 19. Excitation spectra of Zn-MOF-Tb and Zn-MOF-C-Tb. (**a**) Two examples of Zn-MOF-Tb with and without the addition of Cinchonidine and Cinchonine at emission of 544 nm. (**b**) Two examples of Zn-MOF-C-Tb with and without the addition of Cinchonine and Cinchonidine at emission of 544 nm.

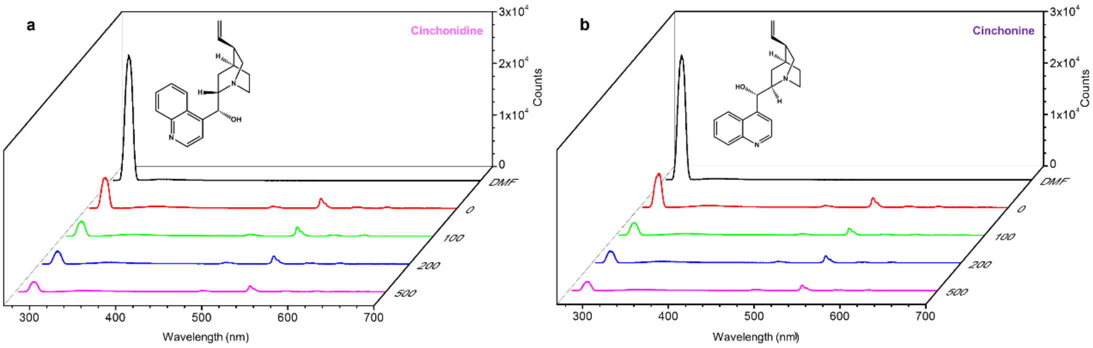

Supplementary Figure 20. Quantum yield spectra. Spectra collected during quantum yield analysis for Zn-MOF-C-Tb dispersed in DMF in the presence of (**a**) Cinchonidine and (**b**) Cinchonine.

Supplementary Table 1. Quantum yield results. Quantum yield results for Zn-MOF-C-Tb in different percentage of Cinchonidine and Cinchonine.

**Additions** [a] **/ mL**	**Quantum yield** [b] **/ %**			
	354 nm		544 nm	
	Cinchonidine	Cinchonine	Cinchonidine	Cinchonine
**0**	6.85	7.04	13.78	14.14
**0.1**	4.95	3.33	10.92	8.34
**0.2**	3.60	2.76	9.31	8.19
**0.5**	1.53	0.49	6.94	6.02

[a] 20 mg additions diluted in 50 mL DMF. [b] Excited at 292 nm.

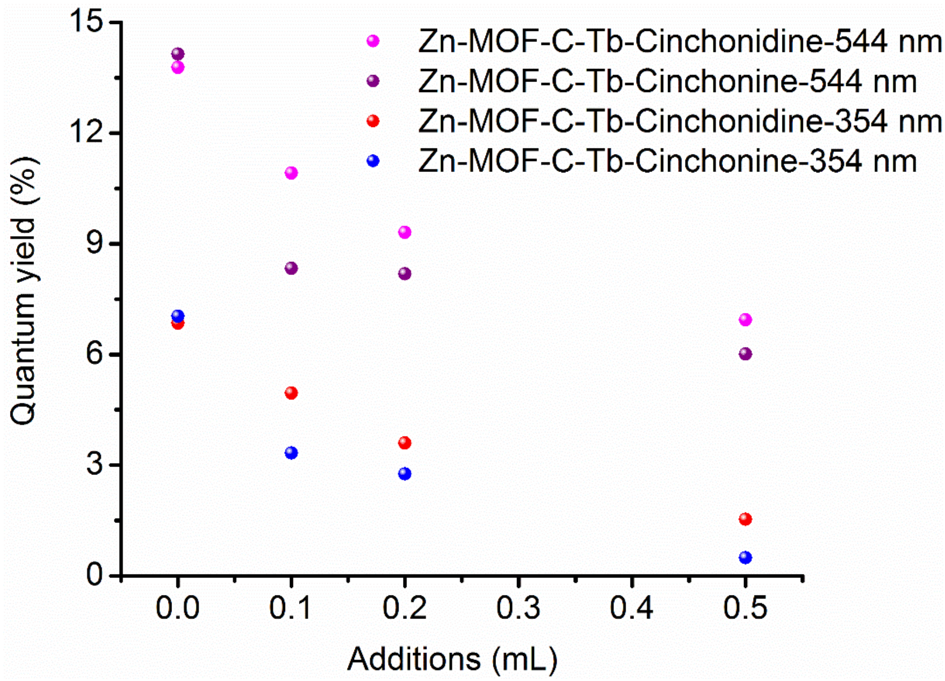

Supplementary Figure 21. Quantum yield patterns. Quantum yield results for Zn-MOF-C-Tb in different percent of Cinchonidine and Cinchonine (2 mg additions diluted in 5 mL DMF) excited at 292 nm.

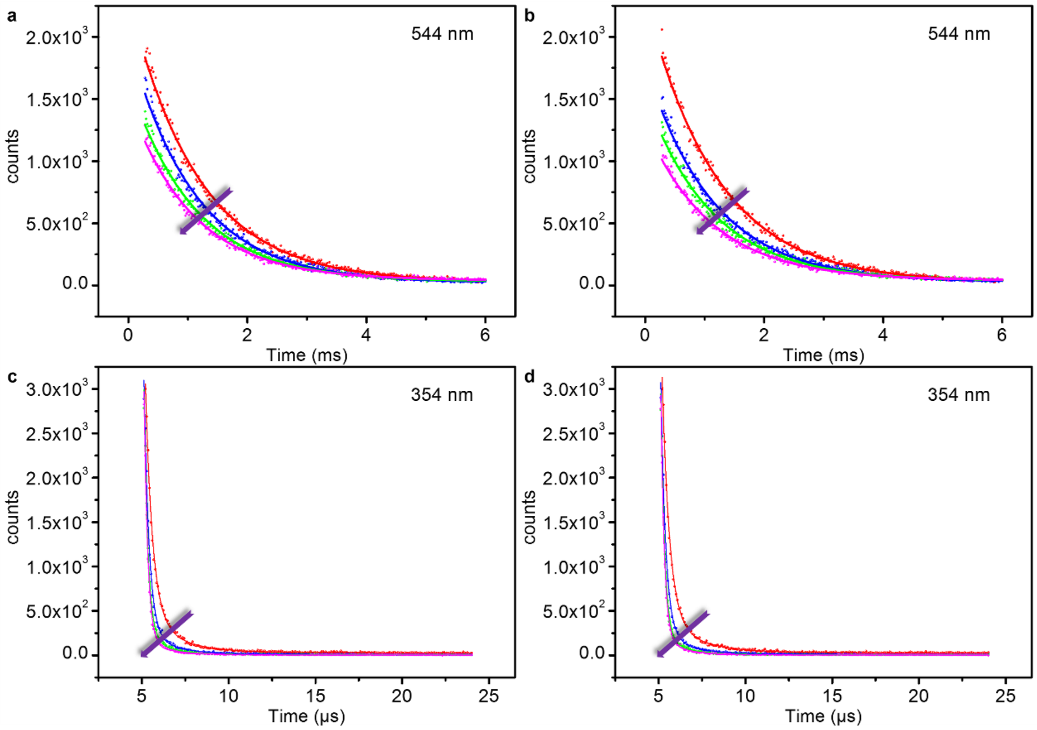

Supplementary Figure 22. Luminescence lifetime patterns. Zn-MOF-C-Tb dispersed in DMF in the presence of (**a**) Cinchonidine at 544 nm, (**b**) Cinchonine at 544 nm and (**c**) Cinchonidine at 354 nm, (**d**) Cinchonine at 354 nm.

Supplementary Table 2. Lifetime fitting results. Zn-MOF-C-Tb in different percent of Cinchonidine and Cinchonine.

**Additions [a] / mL**	**Lifetime [b]**			
	354 nm / ns		544 nm / µs	
	Cinchonidine	Cinchonine	Cinchonidine	Cinchonine
**0**	405.63	416.91	1169.55	1264.41
**0.5**	287.76	279.02	1103.48	1135.72
**1**	226.28	220.08	1091.15	1123.21
**1.5**	215.61	202.49	1071.28	1118.72

[a] 2 mg additions diluted in 5 mL DMF. [b] Excited at 292 nm.

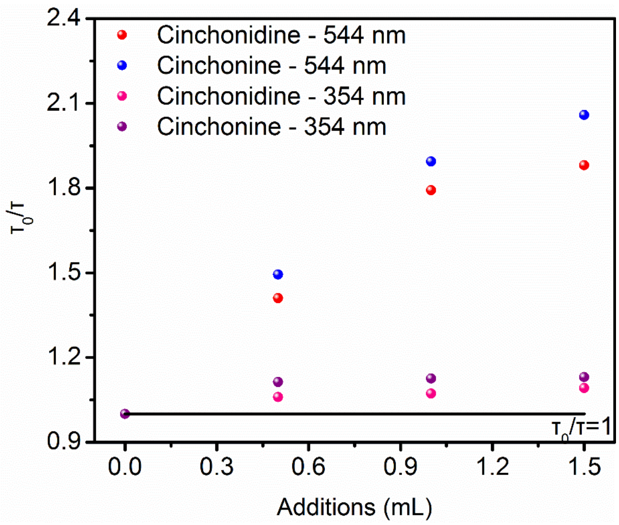

Supplementary Figure 23. Lifetime fitting results. Zn-MOF-C-Tb in different percent of Cinchonidine and Cinchonine. 2 mg additions were diluted in 5 mL DMF.

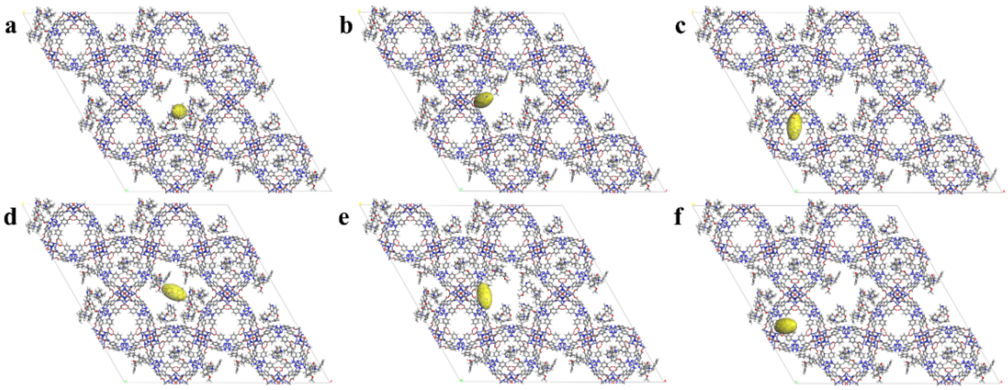

Supplementary Figure 24. Calculated alignment modes for Cinchonine and Cinchonidine in Zn-MOF-C. (abc) are the positions for Zn-MOF-C with Cinchonine and **(def)** are for Zn-MOF-C with Cinchonidine.

Supplementary Table 3. Energy calculations. Energy variation of Zn-MOF-C with Cinchonine and Cinchonidine at different positions.

Positions	*ΔE* (Cinchonine) / kJ·mol^{-1}	*ΔE* (Cinchonidine) / kJ·mol^{-1}
1	-65.30	-18.78
2	-133.30	-126.70
3	-129.28	-109.38

1 stands for position a/d, 2 stands for b/e and 3 stands for c/f in Supplementary Figure 24.

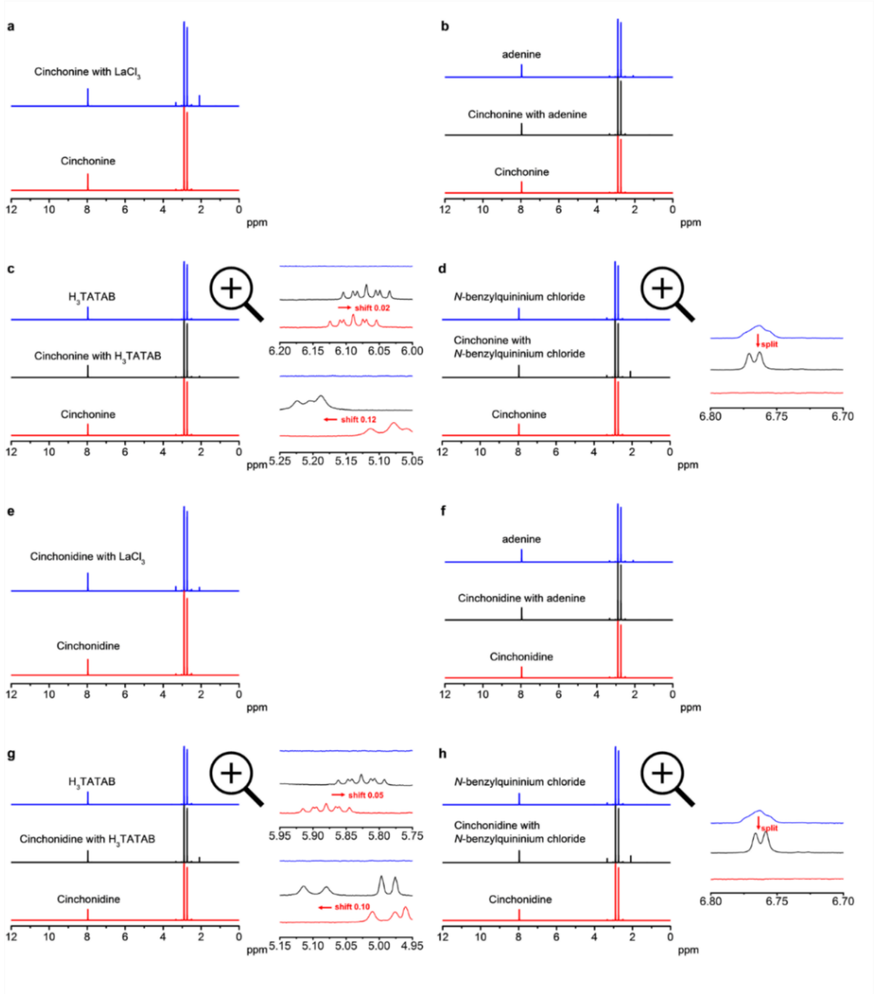

Supplementary Figure 25. Liquid ^{1}H NMR spectra of the analytes and the MOF. DMF solutions of raw materials (10 mM Cinchonine/Cinchonidine, adenine-ligand-1, H_3TATAB-ligand-2, *N*-benzylquininium chloride) and DMF solutions of the mixtures of ligands/chiral center/La^{3+} and epimers (10 mM adenine and Cinchonine/Cinchonidine, H_3TATAB and Cinchonine/Cinchonidine, *N*-benzylquininium chloride and Cinchonine/Cinchonidine, $LaCl_3$ and Cinchonine/Cinchonidine). Each 50 μL of these solutions were mixed with 500 μL d_6-DMSO.

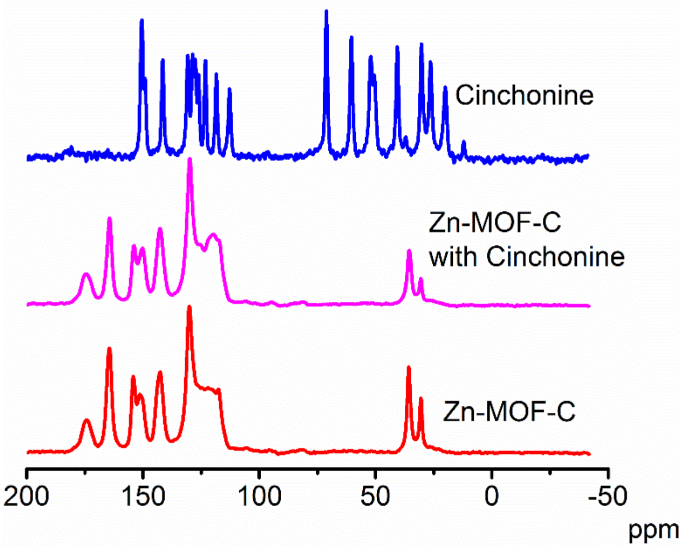

Supplementary Figure 26. Solid-state ^{13}C NMR spectra of the analytes and the MOF. 30 mg Zn-MOF-C was dispersed in 5 mL DMF with 2 mg Cinchonine for 7 days to construct the Zn-MOF-C with Cinchonine.

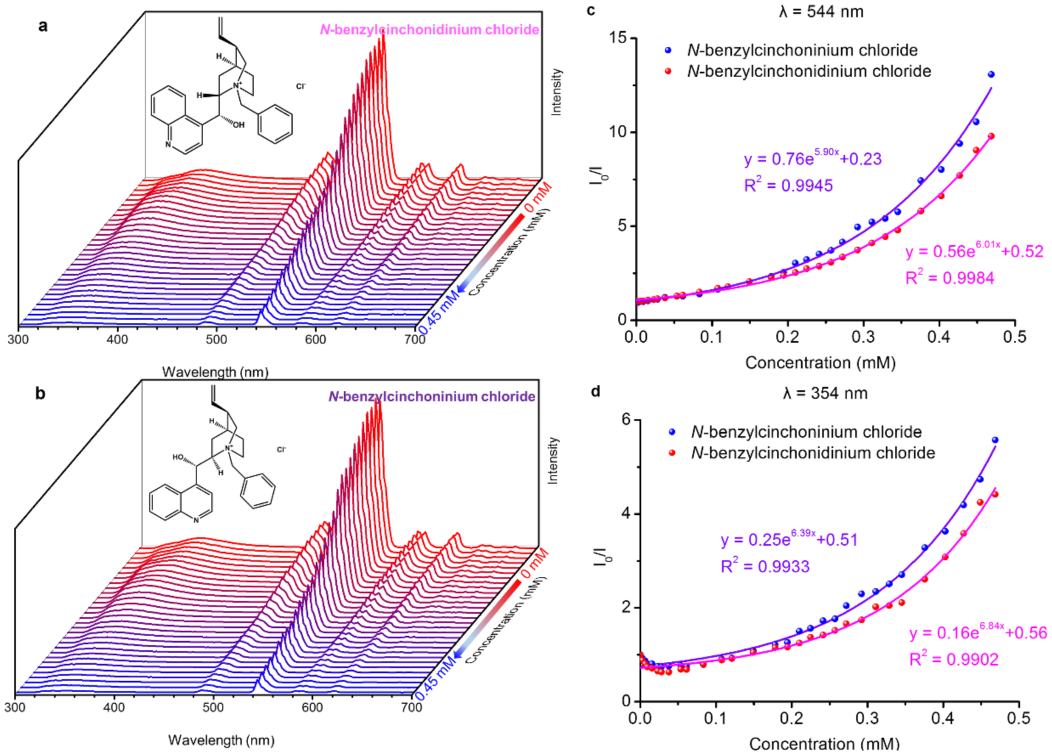

Supplementary Figure 27. Fluorescence emission spectra excited at 292 nm. Zn-MOF-C-Tb dispersed in DMF upon incremental addition of (**a**) *N*-benzylcinchonidinium chloride and (**b**) *N*-benzylcinchoninium chloride. Fluorescence intensity changes of Zn-MOF-C-Tb at (**c**) 544 nm and (**d**) 354 nm.

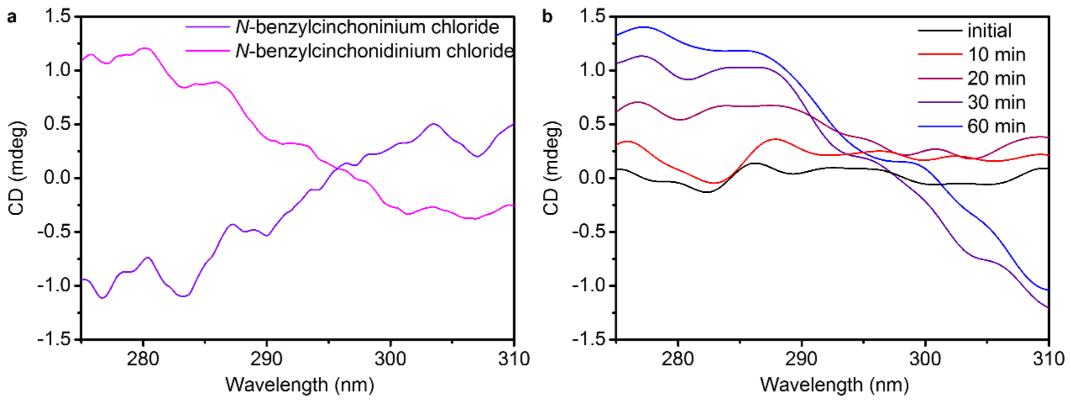

Supplementary Figure 28. Liquid-state CD spectra. (**a**) 10 times dilution of the DMF solution of 0.88 mmol L^{-1} *N*-benzylcinchonidinium chloride and *N*-benzylcinchoninium chloride (2 mg-5 mL) and (**b**) the intensity changes of the equal proportion of the mixture with the addition of Zn-MOF-C-Tb.

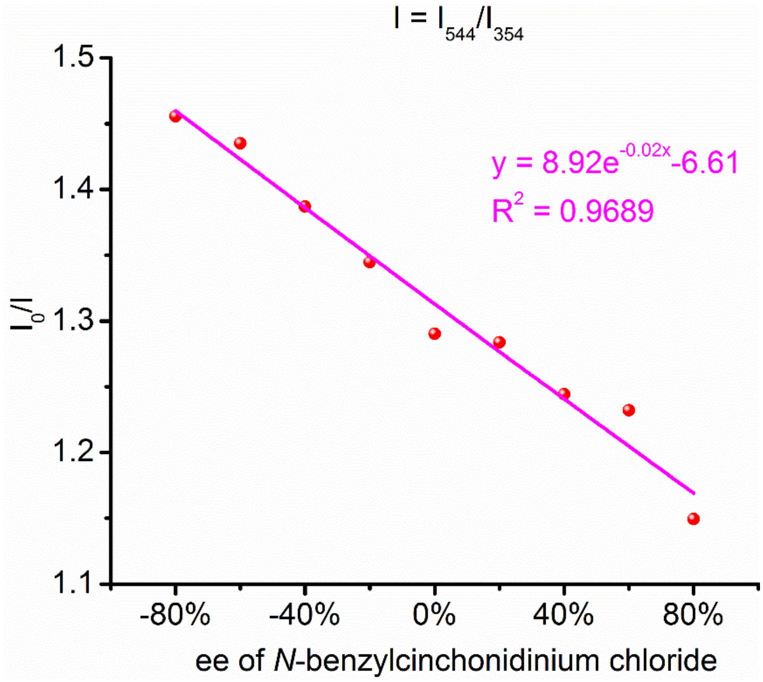

Supplementary Figure 29. *ee* values with fluorescence intensities. Changes of fluorescence intensity of Zn-MOF-C-Tb dispersed in DMF upon incremental addition of 2 mL different percentage of *N*-benzylcinchonidinium chloride and *N*-benzylcinchoninium chloride. I in this figure means I_{544}/I_{354}.

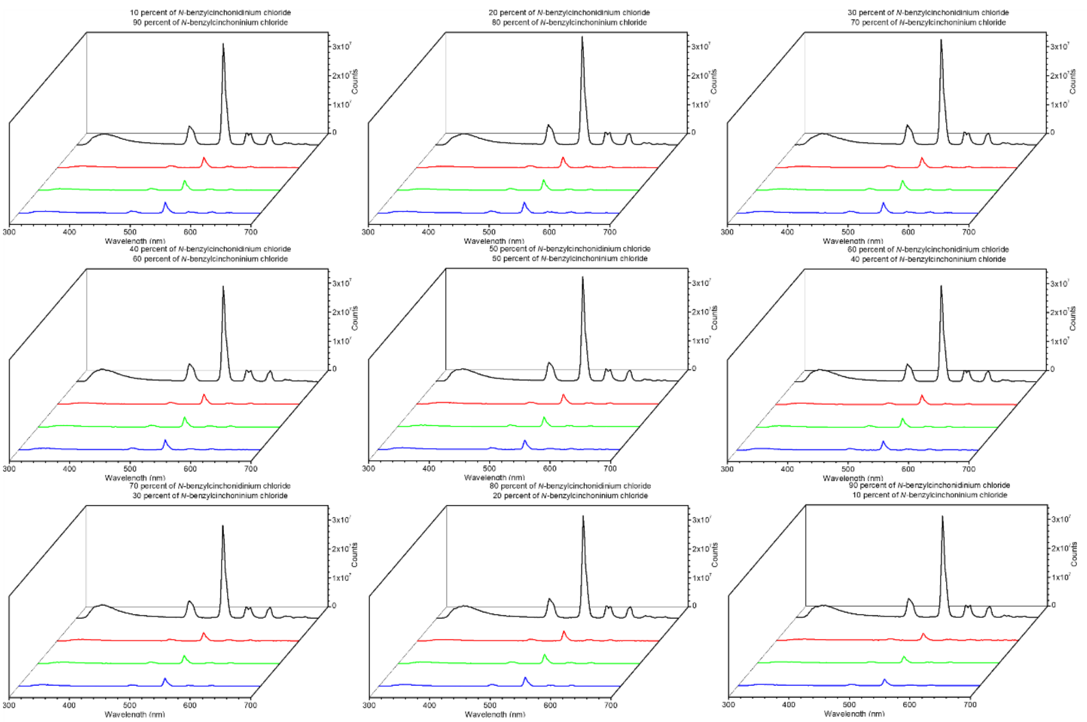

Supplementary Figure 30. Fluorescence intensity changes of Zn-MOF-C-Tb toward addition of a mixture of different proportions of *N*-benzylcinchonidinium chloride and *N*-benzylcinchoninium chloride. Black lines represent the original Zn-MOF-C-Tb while others stand for Zn-MOF-C-Tb with the mixtures of different *ee* values for three times.

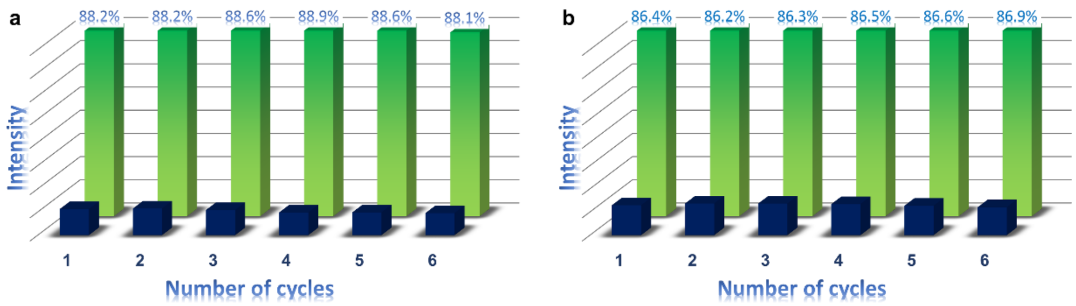

Supplementary Figure 31. Recycling experiment. Quenching ability of Zn-MOF-C-Tb dispersed in DMF in the presence of (**a**) *N*-benzylcinchoninium chloride and (**b**) *N*-benzylcinchonidinium chloride with six cycles (the green and blue bars represent the initial luminescence intensity and the intensity with 1 mmol L^{-1} analytes, respectively) at 544 nm. Numbers above the bars are the quantitative quenching.

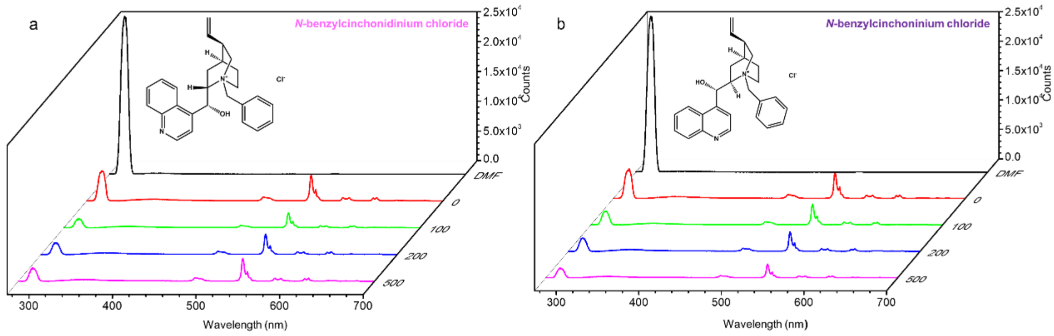

Supplementary Figure 32. Quantum yield spectra. Spectra collected during quantum yield analysis for Zn-MOF-C-Tb dispersed in DMF in the presence of (**a**) *N*-benzylcinchonidinium chloride and (**b**) *N*-benzylcinchoninium chloride.

Supplementary Table 4. Quantum yield results. Quantum yield results for Zn-MOF-C-Tb in different percent of *N*-benzylcinchonidinium chloride and *N*-benzylcinchoninium chloride.

**Additions [a] / mL**	**Quantum yield [b] / %**			
	354 nm		544 nm	
	N-benzylcinchonidinium chloride	*N*-benzylcinchoninium chloride	*N*-benzylcinchonidinium chloride	*N*-benzylcinchoninium chloride
**0**	5.39	5.61	21.14	21.30
**0.1**	4.35	4.65	16.24	15.15
**0.2**	3.95	4.27	14.41	14.29
**0.5**	2.63	2.50	10.10	9.29

[a] 20 mg additions diluted in 50 mL DMF. [b] Excited at 292 nm.

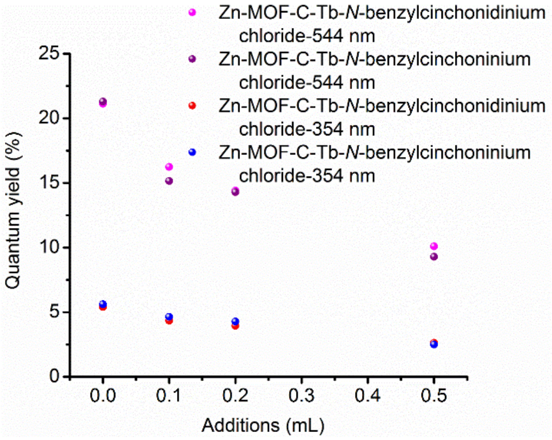

Supplementary Figure 33. Quantum yield patterns. Quantum yield results for Zn-MOF-C-Tb in different percent of *N*-benzylcinchonidinium chloride and *N*-benzylcinchoninium chloride (2 mg additions diluted in 5 mL DMF) excited at 292 nm.

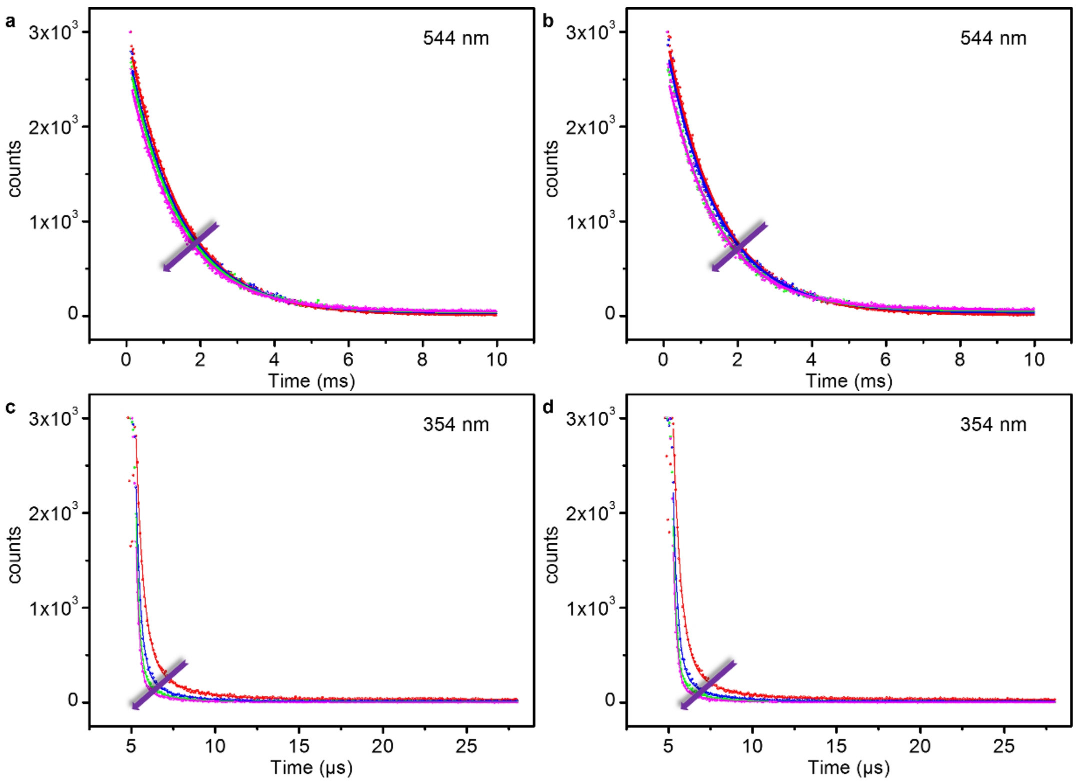

Supplementary Figure 34. Luminescence lifetime patterns. Zn-MOF-C-Tb dispersed in DMF in the presence of (**a**) *N*-benzylcinchonidinium chloride at 544 nm, (**b**) *N*-benzylcinchoninium chloride at 544 nm and (**c**) *N*-benzylcinchonidinium chloride at 354 nm, (**d**) *N*-benzylcinchoninium chloride at 354 nm.

Supplementary Table 5. Lifetime fitting results. Zn-MOF-C-Tb in different percent of *N*-benzylcinchonidinium chloride and *N*-benzylcinchoninium chloride.

**Additions ^{a} / mL**	**Lifetime ^{b}**			
	354 nm / ns		544 nm / μs	
	N-benzylcinchonidinium chloride	*N*-benzylcinchoninium chloride	*N*-benzylcinchonidinium chloride	*N*-benzylcinchoninium chloride
**0**	453.79	465.29	1418.93	1424.44
**0.5**	291.04	278.39	1408.99	1407.78
**1**	218.86	217.87	1377.19	1369.30
**1.5**	211.02	210.69	1362.06	1342.52

^{a} 2 mg additions diluted in 5 mL DMF. ^{b} Excited at 292 nm.

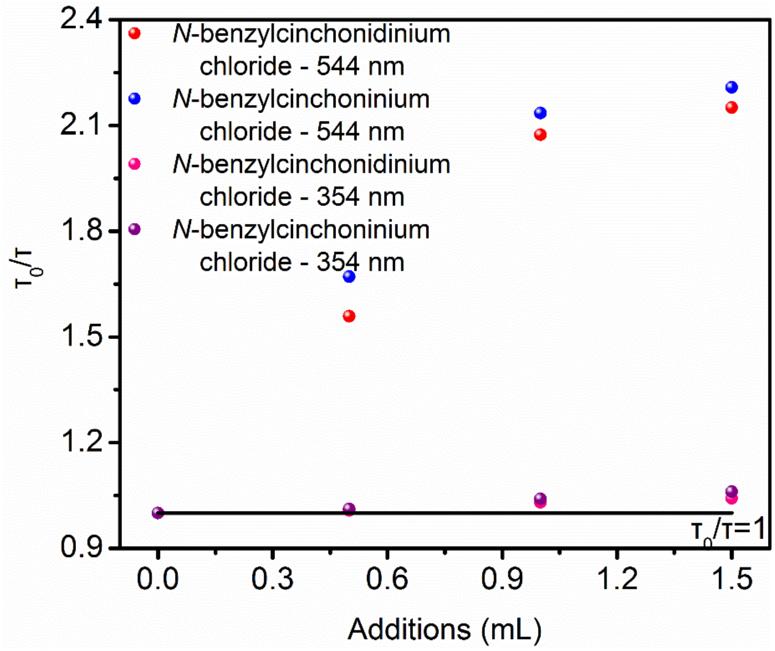

Supplementary Figure 35. Lifetime fitting results. Zn-MOF-C-Tb in different percentage of *N*-benzylcinchonidinium chloride and *N*-benzylcinchoninium chloride. 2 mg additions were diluted in 5 mL DMF.

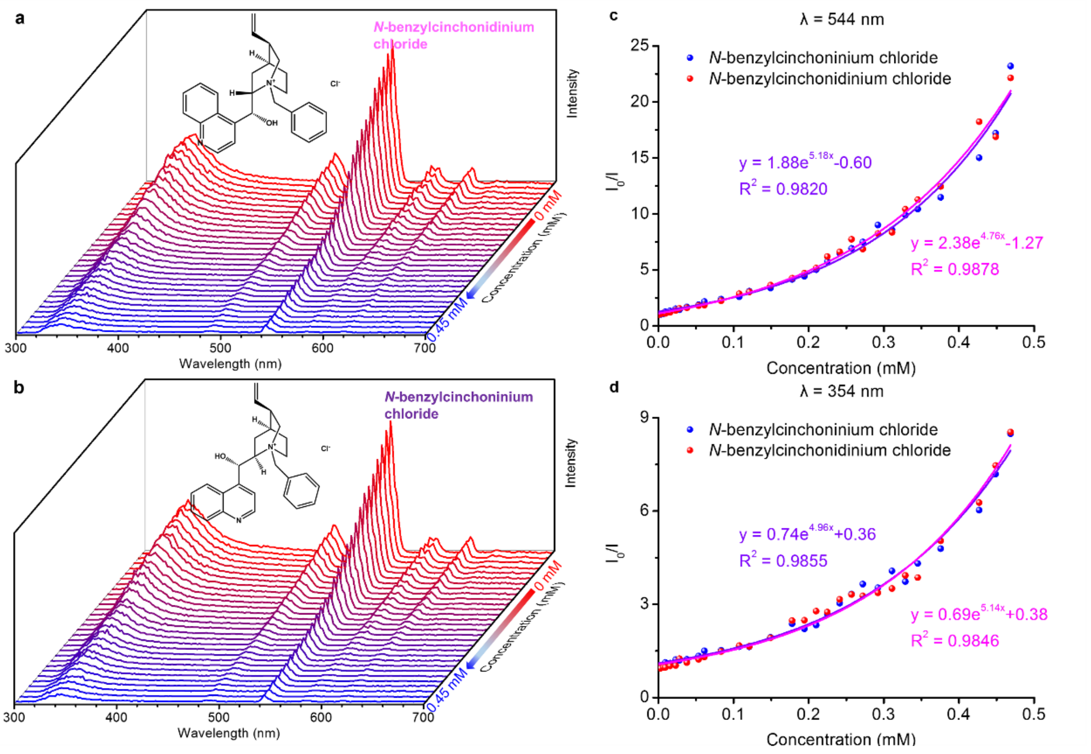

Supplementary Figure 36. Fluorescence emission spectra excited at 292 nm. Zn-MOF-Tb dispersed in DMF upon incremental addition of (**a**) *N*-benzylcinchonidinium chloride and (**b**) *N*-benzylcinchoninium chloride. Fluorescence intensity changes of Zn-MOF-Tb at (**c**) 544 nm and (**d**) 354 nm.

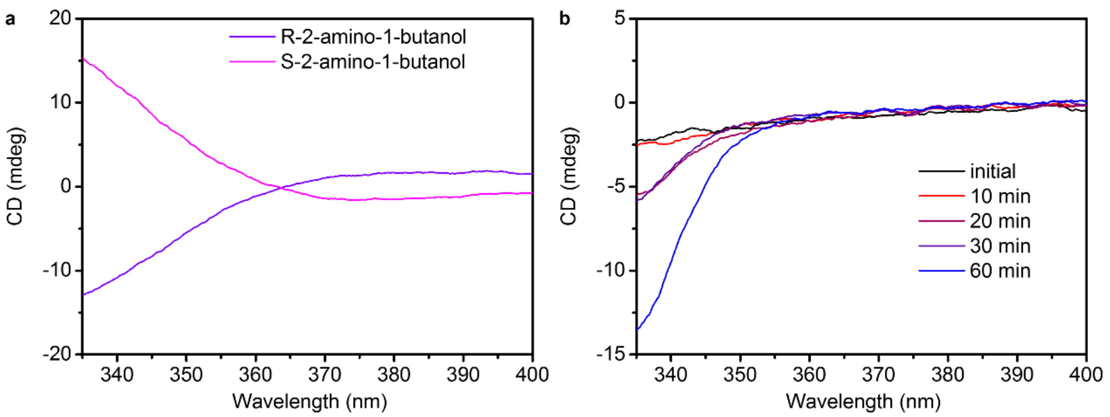

Supplementary Figure 37. Liquid-state CD spectra. (**a**) 5 times dilution of the DMF solution of R-2-amino-1-butanol and S-2-amino-1-butanol and (**b**) the intensity changes of the equal proportion of the mixture with the addition of Zn-MOF-C-Tb.

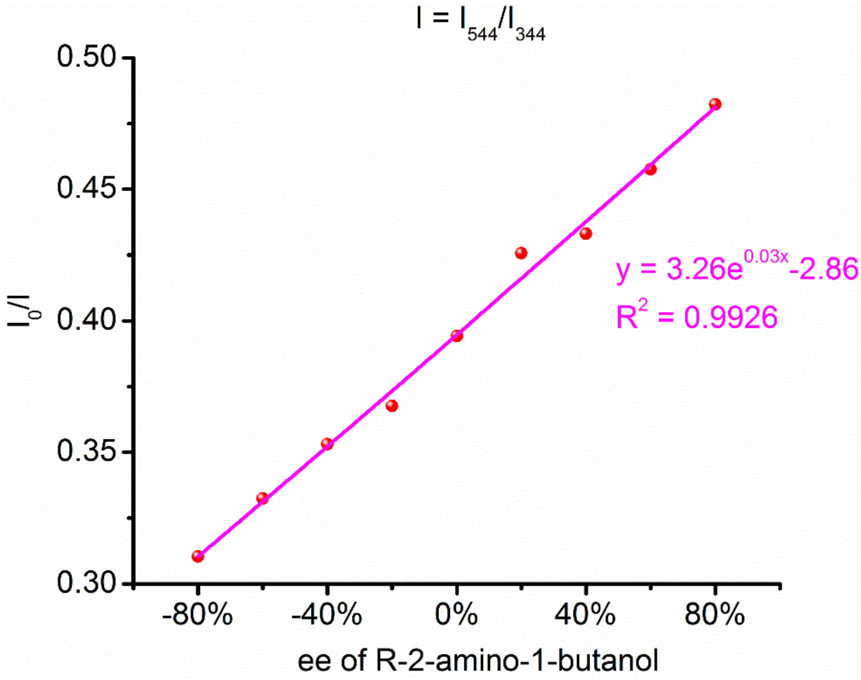

Supplementary Figure 38. *ee* values with fluorescence intensities. Fluorescence intensity changes of Zn-MOF-C-Tb dispersed in DMF upon incremental addition of 0.5 mL different percent of R-2-amino-1-butanol and S-2-amino-1-butanol. I in this figure represents I_{544}/I_{344}.

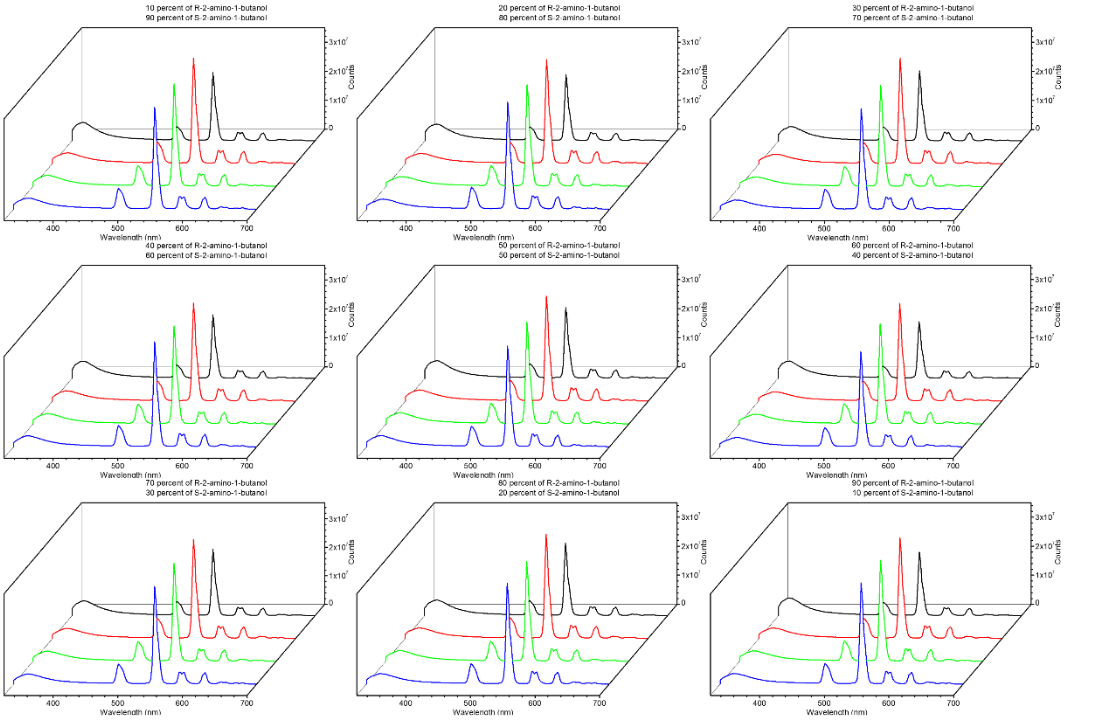

Supplementary Figure 39. Fluorescence intensity changes of Zn-MOF-C-Tb toward addition of a mixture of different proportions of R-2-amino-1-butanol and S-2-amino-1-butanol. Black lines represent the original Zn-MOF-C-Tb while others stand for Zn-MOF-C-Tb with the mixtures of different *ee* values for three times.

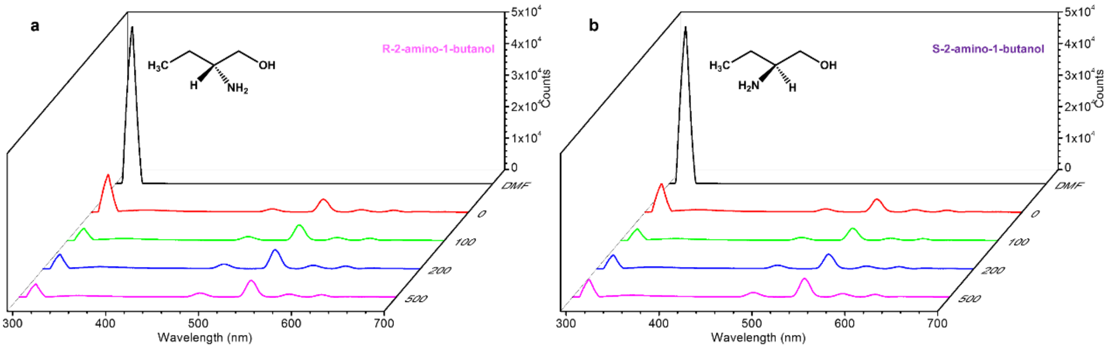

Supplementary Figure 40. Quantum yield spectra. Spectra collected during quantum yield analysis for Zn-MOF-C-Tb dispersed in DMF in the presence of (**a**) R-2-amino-1-butanol and (**b**) S-2-amino-1-butanol.

Supplementary Table 6. Quantum yield results. Quantum yield results for Zn-MOF-C-Tb in different percentage of R-2-amino-1-butanol and S-2-amino-1-butanol.

**Additions [a] / mL**	**Quantum yield [b] / %**			
	344 nm		544 nm	
	R-2-amino-1-butanol	S-2-amino-1-butanol	R-2-amino-1-butanol	S-2-amino-1-butanol
**0**	9.32	10.06	23.56	22.15
**0.1**	7.26	7.72	23.26	18.19
**0.2**	6.55	6.93	24.74	22.20
**0.5**	6.42	5.25	28.99	29.98

[a] 20 μL additions diluted in 50 mL DMF. [b] Excited at 314 nm.

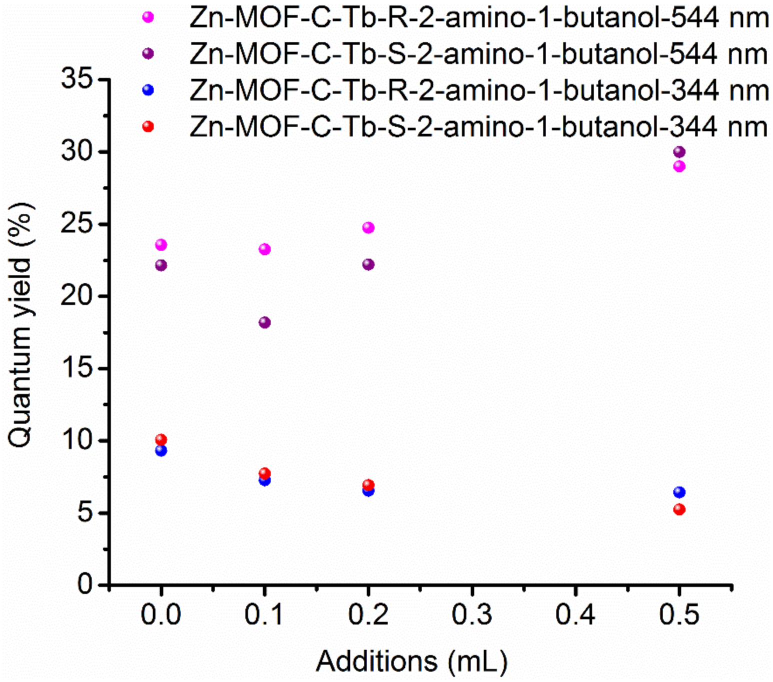

Supplementary Figure 41. Quantum yield patterns. Quantum yield results for Zn-MOF-C-Tb in different percent of R-2-amino-1-butanol and S-2-amino-1-butanol (2 μL additions diluted in 5 mL DMF) excited at 314 nm.

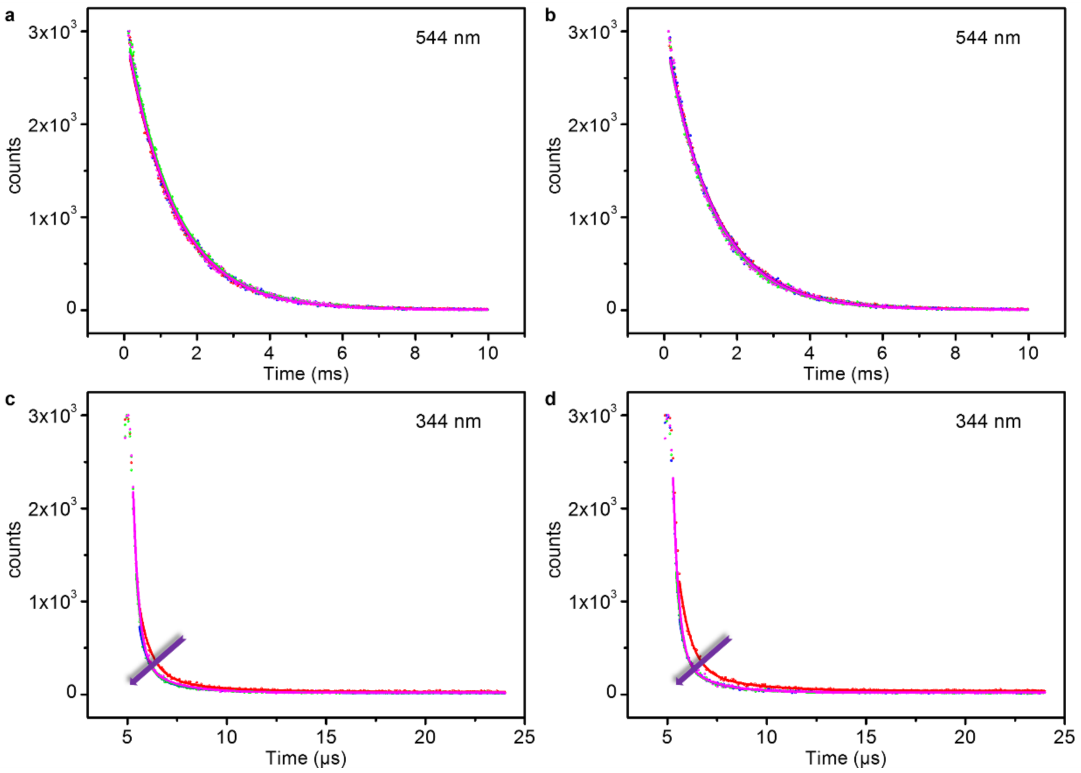

Supplementary Figure 42. Luminescence lifetime patterns. Zn-MOF-C-Tb dispersed in DMF in the presence of (**a**) R-2-amino-1-butanol at 544 nm, (**b**) S-2-amino-1-butanol at 544 nm and (**c**) R-2-amino-1-butanol at 344 nm, (**d**) S-2-amino-1-butanol at 344 nm.

Supplementary Table 7. Lifetime fitting results. Zn-MOF-C-Tb in different percent of R-2-amino-1-butanol and S-2-amino-1-butanol.

**Additions [a] / mL**	**Lifetime [b]**			
	344 nm / ns		544 nm / µs	
	R-2-amino-1-butanol	S-2-amino-1-butanol	R-2-amino-1-butanol	S-2-amino-1-butanol
**0**	602.81	598.33	1323.23	1316.88
**0.5**	447.62	405.22	1331.75	1310.44
**1**	332.60	318.67	1333.21	1305.94
**1.5**	296.60	289.63	1329.85	1313.29

[a] 2 µL additions diluted in 5 mL DMF. [b] Excited at 314 nm.

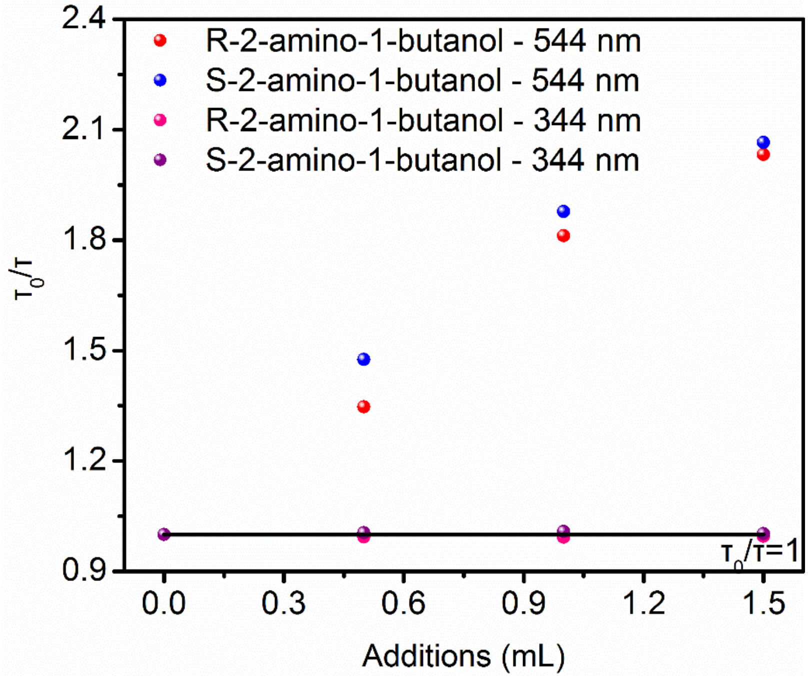

Supplementary Figure 43. Lifetime fitting results. Zn-MOF-C-Tb in different percentage of R-2-amino-1-butanol and S-2-amino-1-butanol. 2 μL additions were diluted in 5 mL DMF.

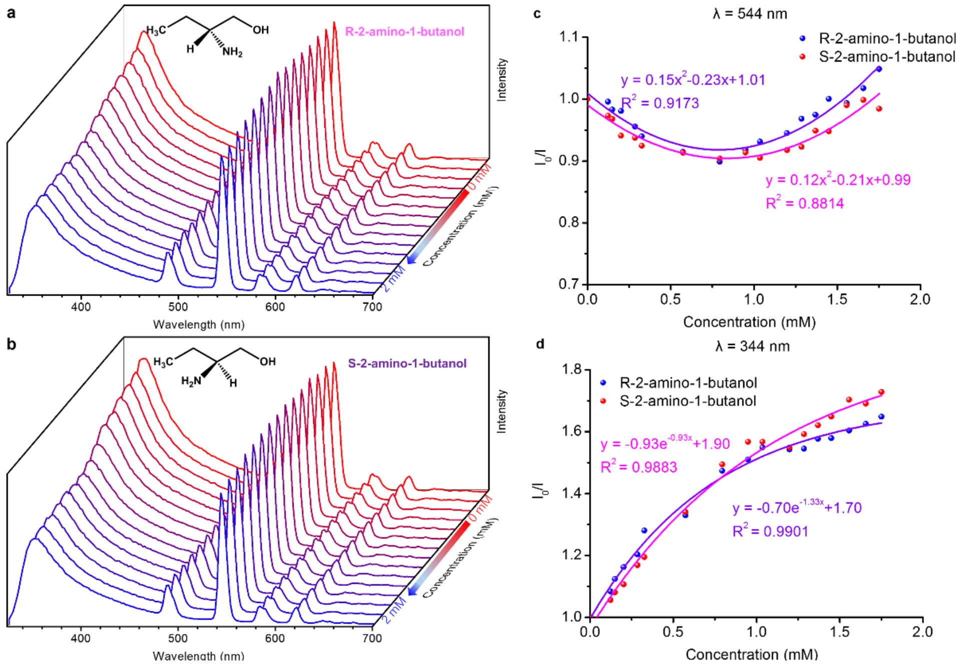

Supplementary Figure 44. Fluorescence emission spectra excited at 314 nm. Zn-MOF-Tb dispersed in DMF upon incremental addition of (**a**) R-2-amino-1-butanol and (**b**) S-2-amino-1-butanol. Fluorescence intensity changes of Zn-MOF-Tb at (**c**) 544 nm and (**d**) 344 nm.

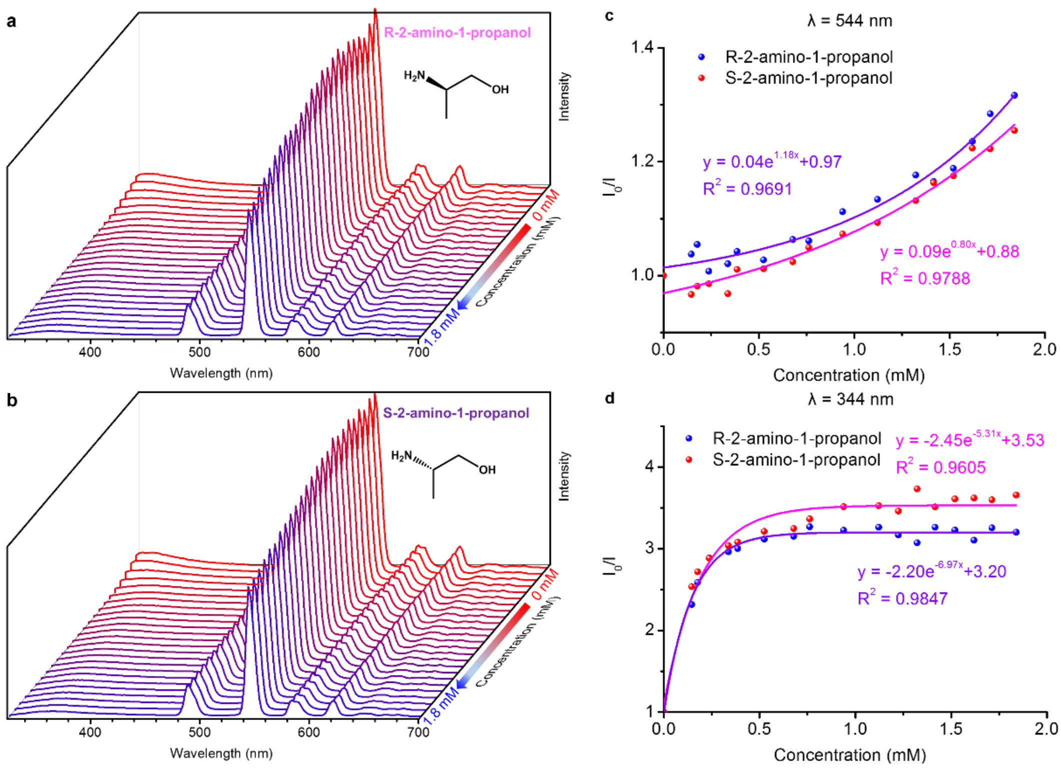

Supplementary Figure 45. Fluorescence emission spectra excited at 314 nm. Zn-MOF-C-Tb dispersed in DMF upon incremental addition of (**a**) R-2-amino-1-propanol and (**b**) S-2-amino-1-propanol. Fluorescence intensity changes of Zn-MOF-C-Tb at (**c**) 544 nm and (**d**) 344 nm.

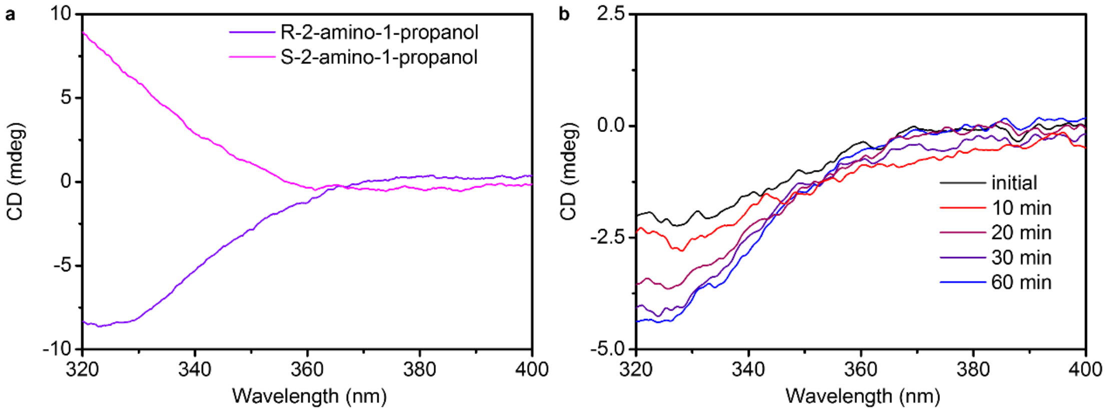

Supplementary Figure 46. Liquid-state CD spectra. (**a**) 5 times dilution of the DMF solution of R-2-amino-1-propanol and S-2-amino-1-propanol and (**b**) the intensity changes of the equal proportion of the mixture with the addition of Zn-MOF-C-Tb.

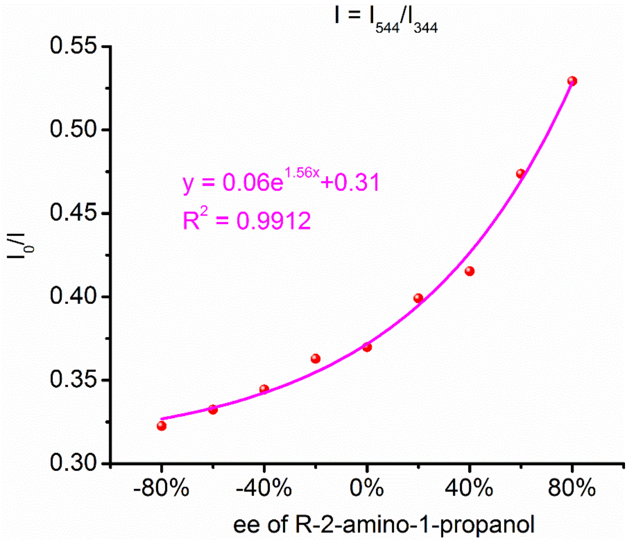

Supplementary Figure 47. *ee* values with fluorescence intensities. Fluorescence intensity changes of Zn-MOF-C-Tb dispersed in DMF upon incremental addition of 0.5 mL different percentage of R-2-amino-1-propanol and S-2-amino-1-propanol. *I* in this figure represents I_{544}/I_{344}.

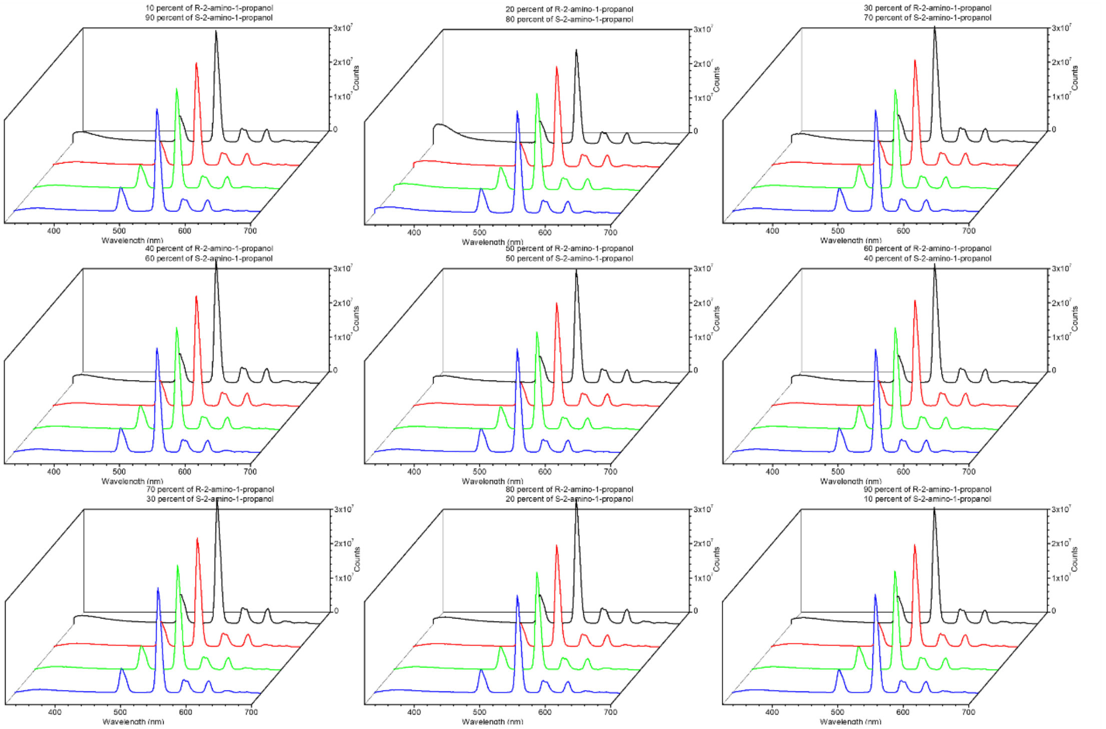

Supplementary Figure 48. Fluorescence intensity changes of Zn-MOF-C-Tb toward addition of a mixture of different proportions of R-2-amino-1-propanol and S-2-amino-1-propanol. Black lines represent the original Zn-MOF-C-Tb while others stand for Zn-MOF-C-Tb with the mixtures of different *ee* values for three times.

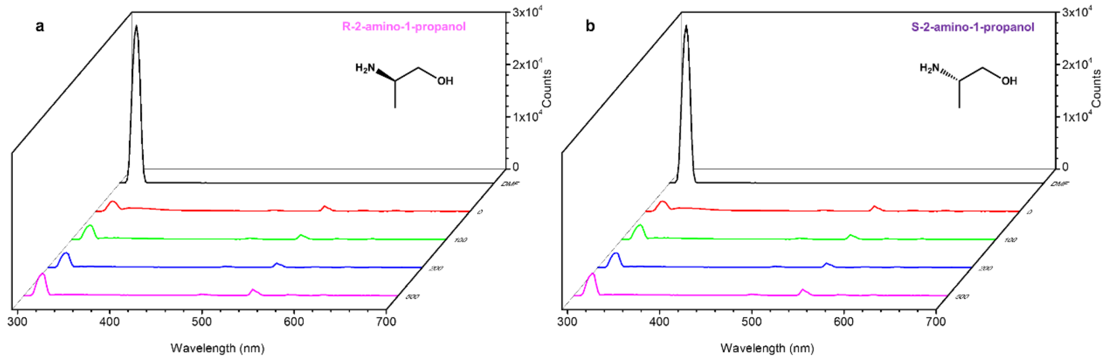

Supplementary Figure 49. Quantum yield spectra. Spectra collected during quantum yield analysis for Zn-MOF-C-Tb dispersed in DMF in the presence of (**a**) R-2-amino-1-propanol and (**b**) S-2-amino-1-propanol.

Supplementary Table 8. Quantum yield results. Quantum yield results for Zn-MOF-C-Tb in different percentage of R-2-amino-1-propanol and S-2-amino-1-propanol.

**Additions [a] / mL**	**Quantum yield [b] / %**			
	344 nm		544 nm	
	R-2-amino-1-propanol	S-2-amino-1-propanol	R-2-amino-1-propanol	S-2-amino-1-propanol
**0**	8.43	8.18	17.45	17.77
**0.1**	3.56	3.24	16.45	16.23
**0.2**	2.55	2.23	14.62	14.47
**0.5**	2.47	2.05	13.46	12.42

[a] 20 μL additions diluted in 50 mL DMF. [b] Excited at 314 nm.

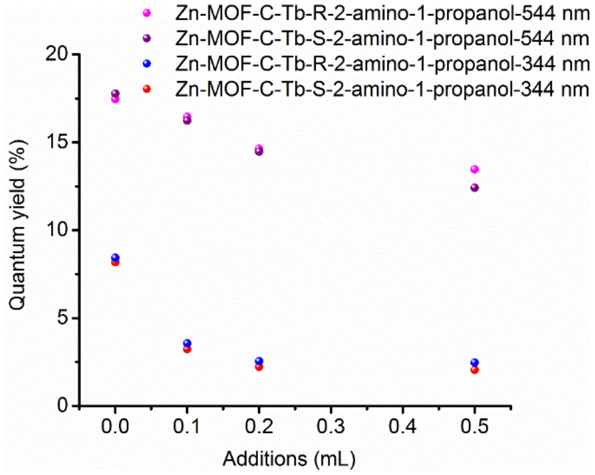

Supplementary Figure 50. Quantum yield patterns. Quantum yield results for Zn-MOF-C-Tb in different percent of R-2-amino-1-propanol and S-2-amino-1-propanol (2 μL additions diluted in 5 mL DMF) excited at 314 nm.

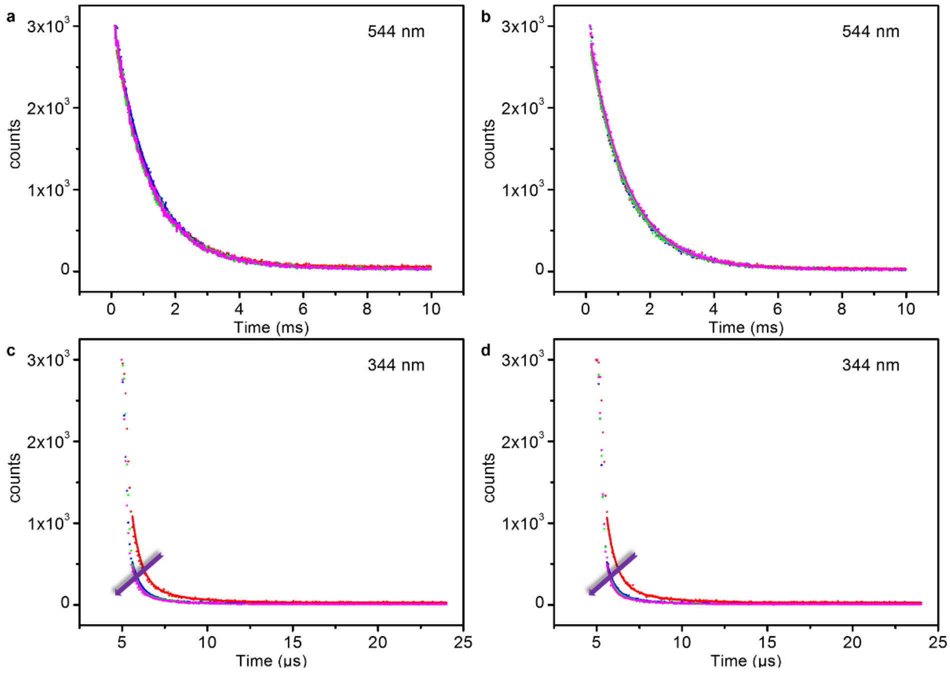

Supplementary Figure 51. Luminescence lifetime patterns. Zn-MOF-C-Tb dispersed in DMF in the presence of (**a**) R-2-amino-1-propanol at 544 nm, (**b**) S-2-amino-1-propanol at 544 nm and (**c**) R-2-amino-1-propanol at 344 nm, (**d**) S-2-amino-1-propanol at 344 nm.

Supplementary Table 9. Lifetime fitting results. Zn-MOF-C-Tb in different percent of R-2-amino-1-propanol and S-2-amino-1-propanol.

**Additions** ^{a} **/ mL**	**Lifetime** ^{b}			
	344 nm / ns		544 nm / μs	
	R-2-amino-1-propanol	S-2-amino-1-propanol	R-2-amino-1-propanol	S-2-amino-1-propanol
**0**	522.31	538.02	1172.01	1170.86
**0.5**	512.51	480.31	1171.57	1171.27
**1**	430.56	443.05	1166.77	1172.81
**1.5**	377.60	323.13	1164.03	1180.14

^{a} 2 μL additions diluted in 5 mL DMF. ^{b} Excited at 314 nm.

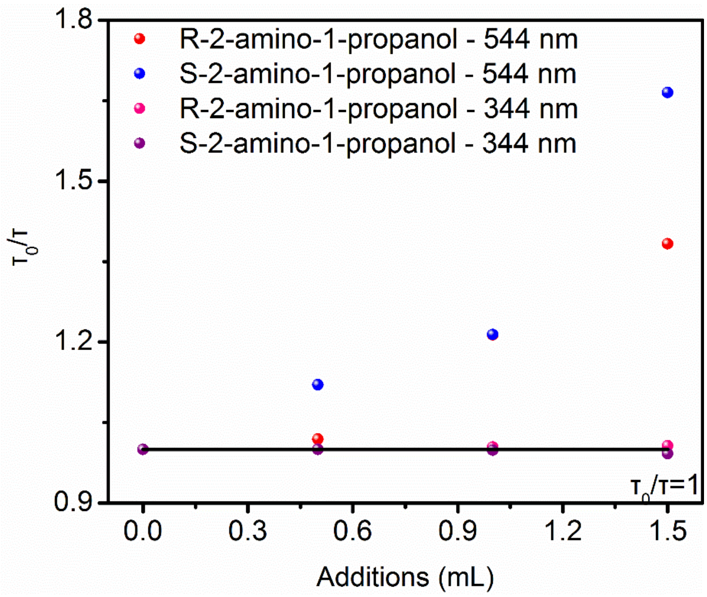

Supplementary Figure 52. Lifetime fitting results. Zn-MOF-C-Tb in different percentage of R-2-amino-1-propanol and S-2-amino-1-propanol. 2 μL additions were diluted in 5 mL DMF.

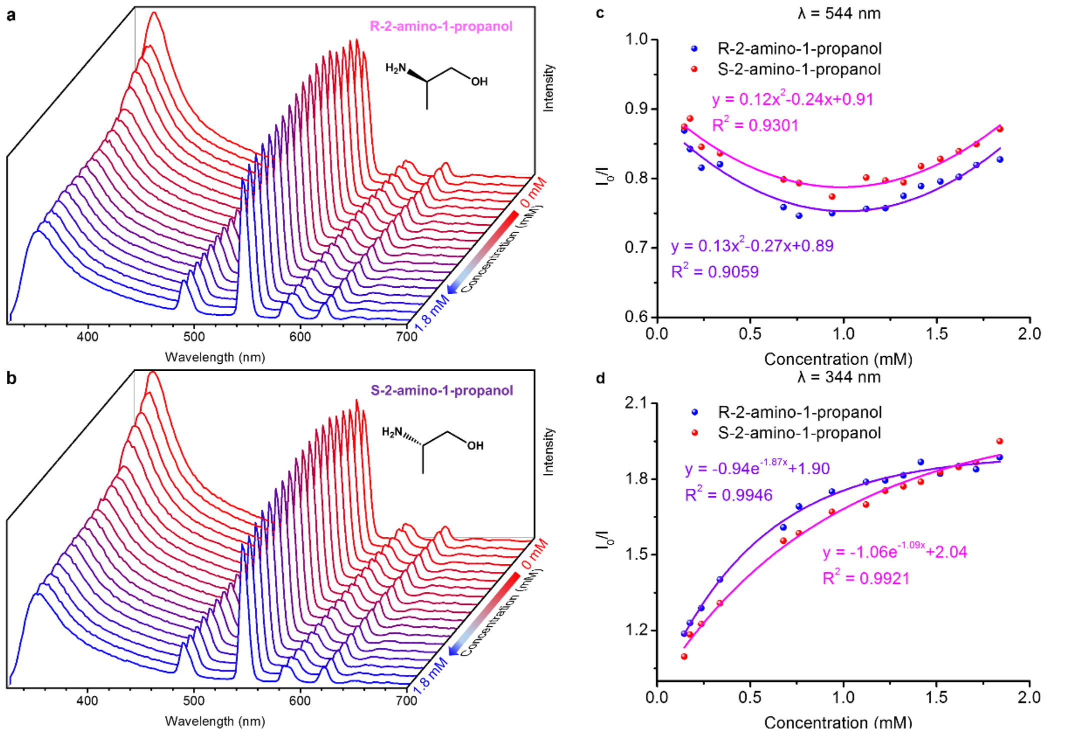

Supplementary Figure 53. Fluorescence emission spectra excited at 314 nm. Zn-MOF-Tb dispersed in DMF upon incremental addition of (**a**) R-2-amino-1-propanol and (**b**) S-2-amino-1-propanol. Fluorescence intensity changes of Zn-MOF-Tb at (**c**) 544 nm and (**d**) 344 nm.

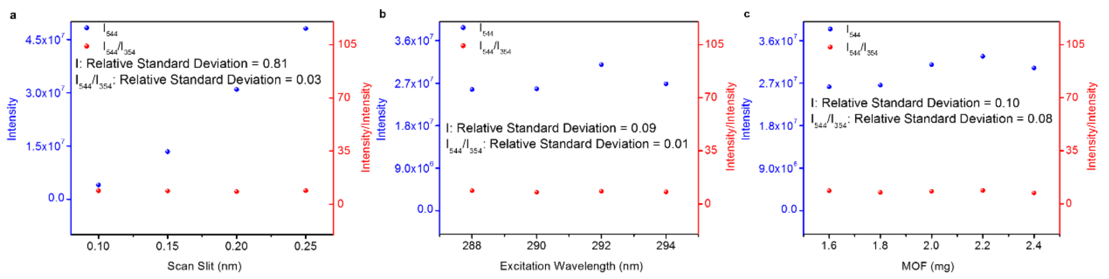

Supplementary Figure 54. Interference experiments. Fluorescence intensities of Zn-MOF-C-Tb dispersed in DMF at (**a**) different scan slits (**b**) different excitation wavelengths (**c**) different concentrations of MOFs at 544 nm and the ratio of 544 nm and 354 nm.

Supplementary Table 10. Enantioselective recognition in literature. Summary of enantioselective luminescence recognition by coordination compounds.

coordination compounds	categories	analytes	K_{SV}	K_{BH}	K_{SV1}/K_{SV2} (K_{BH1}/K_{BH2})	centers	references
**$[Cl(CO)_3Re(L_1)]_4$**	supramolecule	2-amino-1-propanol	7.35(S)<br>6.02(R)		1.22	ligand	1
**$\{[Cd_2(L_2)(H_2O)_2]\cdot 6.5DMF\cdot 3EtOH\}_n$**	MOF	2-amino-1-propanol	19.4×10^3(S)<br>15.5×10^3(R)		1.25	ligand	2
		2-amino-2-phenylethanol	31×10^3(S)<br>27×10^3(R)		1.17		
		2-amino-3-phenylpropanol	0.68×10^3(S)<br>0.49×10^3(R)		1.39		
		2-amino-3-methyl-1-butanol	1.66×10^3(S)<br>0.53×10^3(R)		3.12		
**$[Zn_8(L_3)_4Cl_8]\cdot 5THF$**	cage	alanine		12.4×10^3(D)<br>3.4×10^3(L)	3.69	ligand	3
**$[Zn_8(L_4)_4I_8]\cdot 4MeOH\cdot 4H_2O$**	cage	1-phenylethylamine			2.87	ligand	4
		1-phenylpropylamine			1.32		
		1-(4-methylphenyl)ethylamine			1.30		
		1-(4-chlorophenyl)ethylamine			1.89		
**$[(Me_2NH_2)Zn_2(L_5)_{1.5}(H_2O)_2]_n$**	MOF	histidine	115(D)<br>64(L)		1.80	ligand	5
**this work**	MOF	Cinchonine	4.66×10^3(Cinchonine)<br>3.45×10^3(Cinchonidine)		1.35	Tb	
			2.91×10^3(Cinchonine)<br>2.37×10^3(Cinchonidine)		1.23	ligand	
		N-benzylcinchoninium chloride	4.48×10^3(*N*-benzylcinchoninium chloride)<br>3.37×10^3(*N*-benzylcinchonidinium chloride)		1.33	Tb	
			1.60×10^3(*N*-benzylcinchoninium chloride)<br>1.09×10^3(*N*-benzylcinchonidinium chloride)		1.46	ligand	
		2-amino-1-butanol		5.56×10^3(R)<br>2.57×10^3(S)	2.16	Tb	
			0.62×10^3(S)<br>0.18×10^3(R)		3.45	ligand	
		2-amino-1-propanol	72(S)<br>47(R)		1.53	Tb	
			15.33×10^3(S)<br>13.01×10^3(R)		1.18	ligand	

L_1: 6,6'-dichloro-2,2'-dihydroxy-4,4'-bis(4-pyridyl)-1,1'-binaphthalene; L_2: 2,2'-dihydroxy-1,1'-binaphthyl-4,4',6,6'-tetrakis(4-benzoic acid); L_3: *N,N'*-bis(3-(*E*)-4-vinylpyridy-5-*tert*-butyl-2-hydroxybenzyl)-trans-(*R,R*)-1,2-diaminocyclohexane; L_4: *N,N'*-bis(3-*tert*-butyl-5-(4-pyridyl)salicylidene)-1,2-diaminocyclo-hexane); L_5: pyrene-tetralactic acid. K_{SV} is the quenching constant from the Stern-Volmer equation and K_{BH} is the association constant from the Benesi-Hildebrand equation. For linear quenching-type, linear Stern-Volmer equation $I_0/I = 1+K_{SV}[C]$. For nonlinear quenching-type, nonlinear Stern-Volmer equation $I_0/I = a\cdot\exp(k[C])+b$, K_{SV} is calculated from a and k.[6,7,8] For enhancement-type, Benesi-Hildebrand equation $I_0/(I-I_0) = K_{BH}/[C]$.[3,9,10]

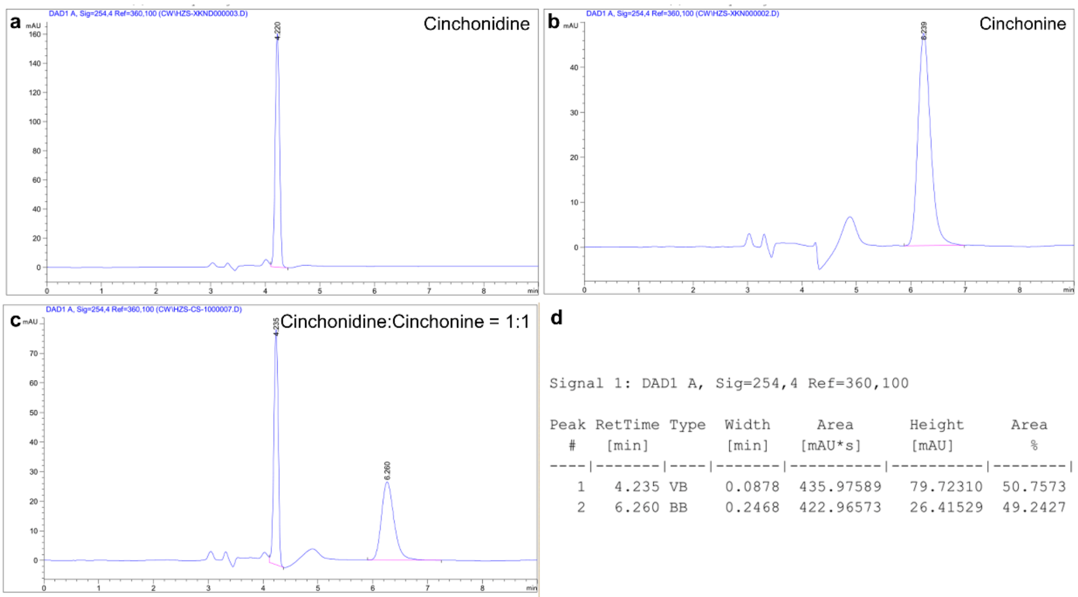

Signal 1: DAD1 A, Sig=254,4 Ref=360,100

Peak #	RetTime [min]	Type	Width [min]	Area [mAU*s]	Height [mAU]	Area %
1	4.235	VB	0.0878	435.97589	79.72310	50.7573
2	6.260	BB	0.2468	422.96573	26.41529	49.2427

Supplementary Figure 55. High performance liquid chromatography for Cinchonidine and Cinchonine and the mixture. From the patterns, there is no Cinchonine in Cinchonidine from **a** (no peaks at 6.26) and no Cinchonidine in Cinchonine from **b** (no peaks at 4.24). And the equal proportion mixture shows 1:1 peak area ratio in **c** and **d**. The purities of the analytes are fine.

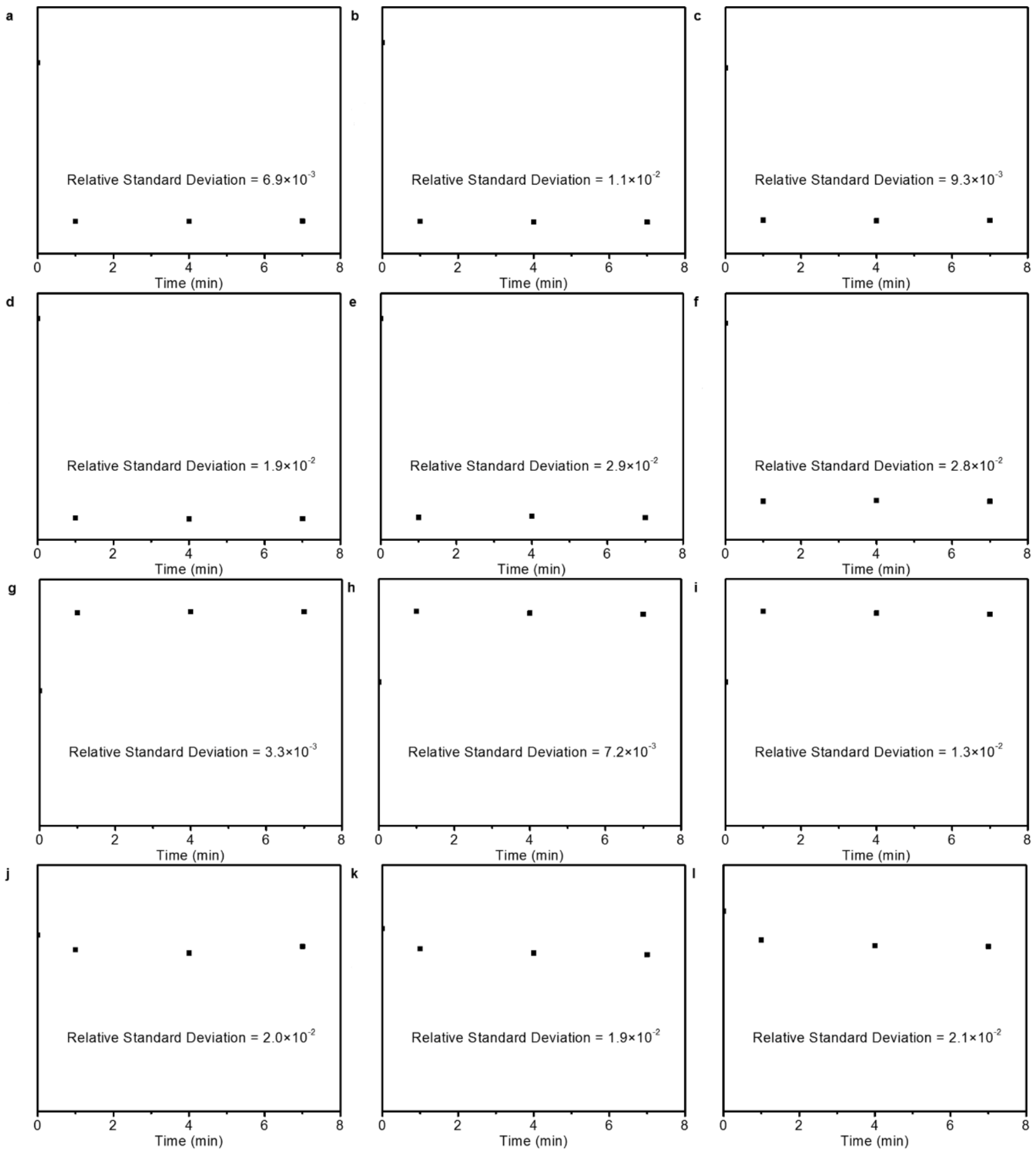

Supplementary Figure 56. Intensity at 544 nm with time. Figures **abc** are the intensity of the peaks at 544 nm of Zn-MOF-C-Tb with Cinchonine. Figures **def** are the intensity of the peaks at 544 nm of Zn-MOF-C-Tb with *N*-benzylcinchoninium chloride. Figures **ghi** are the intensity of the peaks at 544 nm of Zn-MOF-C-Tb with R-2-amino-1-butanol. Figures **jkl** are the intensity of the peaks at 544 nm of Zn-MOF-C-Tb with R-2-amino-1-propanol. The intensity while x is 0 is the original Zn-MOF-C-Tb, the relative standard deviation is calculated by the last 3 points. Each line is tested after one-minute ultrasonic. The peaks changed little after the first minute.

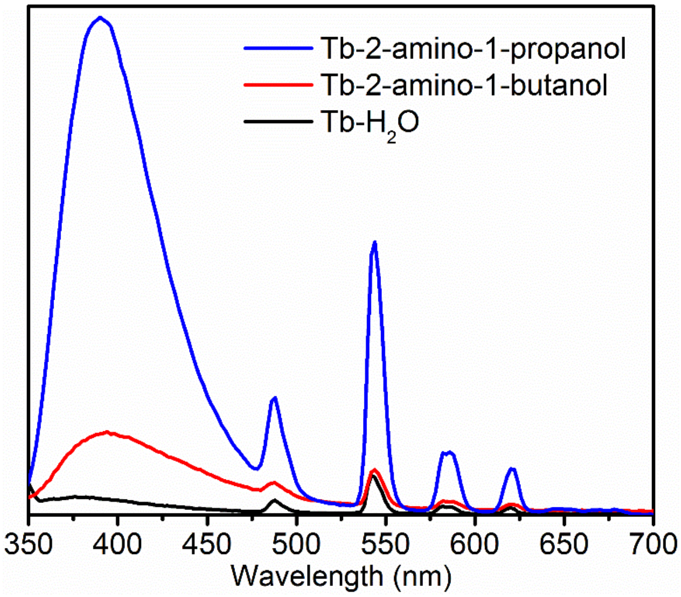

Supplementary Figure 57. Liquid fluorescence spectrum. There were 7 mg of $Tb(OSO_2CF_3)_3$ in 2 mL different solutions.

Supplementary Table 11. HOMO and LUMO of ligands and analytes.

	**HOMO (ev)**	**LUMO (ev)**
**H_3TATAB**	-5.621	-2.467
**Adenine**	-5.054	-1.514
**Cinchonine**	-4.572	-2.311
***N*-benzylcinchoninium chloride**	-8.058	-5.203
**R-2-amino-1-butanol**	-5.062	1.403
**R-2-amino-1-propanol**	-5.097	1.227

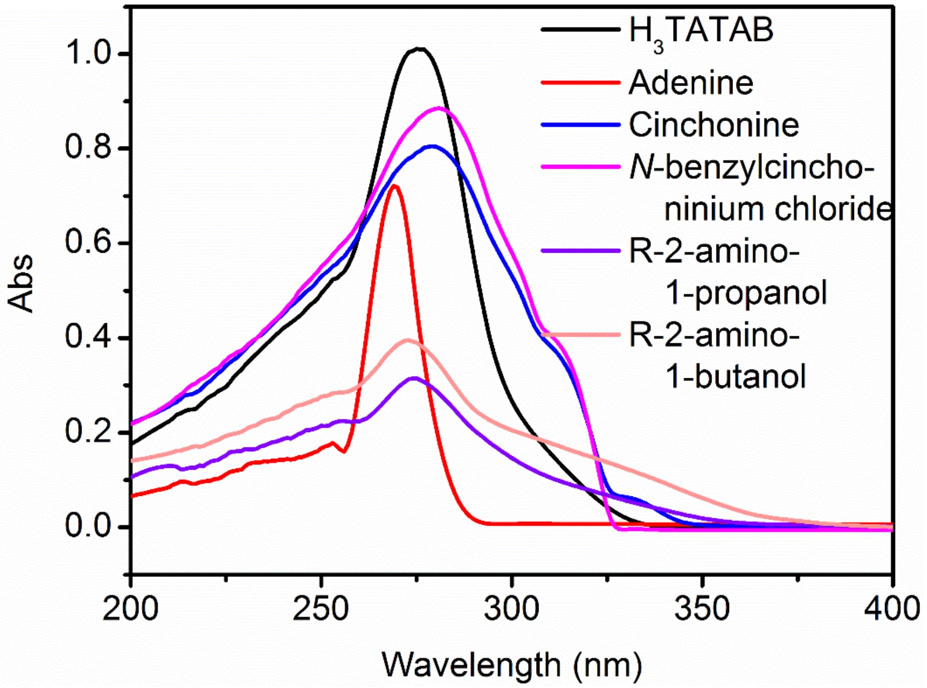

Supplementary Figure 58. Liquid UV-vis spectra. Liquid UV-vis spectra of the ligands of Zn-MOF and analytes in DMF.

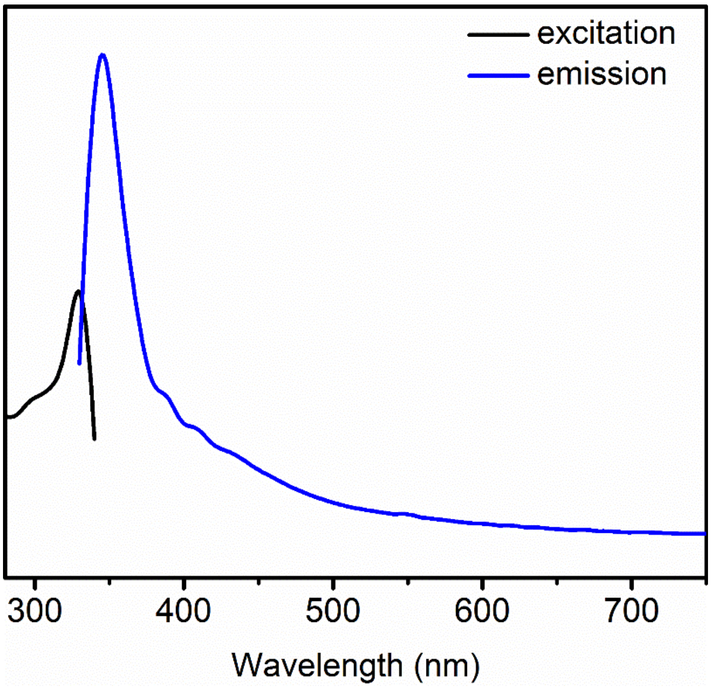

Supplementary Figure 59. Solid-state fluorescence spectra. Solid-state fluorescence spectra for the original Zn-MOF.

Supplementary References

1. Wanderley, M.M., Wang, C., Wu, C.D., Lin, W.B. A chiral porous metal-organic

framework for highly sensitive and enantioselective fluorescence sensing of amino alcohols. *J. Am. Chem. Soc.* **134,** 9050-9053 (2012).

2. Xuan, W., Zhang, M., Liu, Y., Chen, Z., Cui, Y. A chiral quadruple-stranded helicate cage for enantioselective recognition and separation. *J. Am. Chem. Soc.* **134,** 6904-6907 (2012).

3. Dong, J., Zhou, Y., Zhang, F., Cui, Y. A highly fluorescent metallosalalen-based chiral cage for enantioselective recognition and sensing. *Chem. -Eur. J.* **20,** 6455-6461 (2014).

4. Pujari, C., Arindam, M., Govardhan, S., Jarugu, N.M. Remarkably selective and enantiodifferentiating sensing of histidine by a fluorescent homochiral Zn-MOF based on pyrene-tetralactic acid. *Chem. Sci.* **7,** 3085-3091 (2016).

5. Dong, J., et al. Chiral NH-controlled supramolecular metallacycles. *J. Am. Chem. Soc.* **139,** 1554-1564 (2017).

6. Wei, W., Lu, R., Tanga, S., Liu, X. Highly cross-linked fluorescent poly(cyclotriphosphazene-*co*-curcumin) microspheres for the selective detection of picric acid in solution phase. *J. Mater. Chem. A* **3,** 4604-4611 (2015).

7. Sun, X.C., et al. Microwave-assisted ultrafast and facile synthesis of fluorescent carbon nanoparticles from a single precursor: preparation, characterization and their application for the highly selective detection of explosive picric acid. *J. Mater. Chem. A* **4,** 4161-4171 (2016).

8. Wang, L., et al. Detection of polychlorinated benzenes (persistent organic pollutants) by a luminescent sensor based on a lanthanide metal-organic framework. *J. Mater. Chem. A* **5,** 5541-5549 (2017).

9. Arunkumar, E., Ajayaghosh, A., Daub, J. Selective calcium ion sensing with a bichromophoric squaraine foldamer. *J. Am. Chem. Soc.* **127,** 3156-3164 (2005).

10. Chu, Q., Medvetz, D.A., Pang, Y. A polymeric colorimetric sensor with excited-state intramolecular proton transfer for anionic species. *Chem. Mater.* **19,** 6421-6429 (2007).
